# Prognostic landscape of mitochondrial genome in myelodysplastic syndrome after stem-cell transplantation

**DOI:** 10.1186/s13045-023-01418-4

**Published:** 2023-03-10

**Authors:** Jing Dong, Christopher Staffi Buradagunta, Tao Zhang, Stephen Spellman, Yung-Tsi Bolon, Amy E. DeZern, Shahinaz M. Gadalla, H. Joachim Deeg, Aziz Nazha, Corey Cutler, Chao Cheng, Raul Urrutia, Paul Auer, Wael Saber

**Affiliations:** 1 Division of Hematology and Oncology, Department of Medicine, Medical College of Wisconsin, 8701 Watertown Plank Road, HRC 5860, Milwaukee, WI 53226 USA; 2 Medical College of Wisconsin Cancer Center, Milwaukee, WI USA; 3 Linda T. and John A. Mellowes Center for Genomic Sciences and Precision Medicine, Medical College of Wisconsin, Milwaukee, WI USA; 4 CIBMTR® (Center for International Blood and Marrow Transplant Research), National Marrow Donor Program®/Be The Match®, Minneapolis, MN USA; 5 The Sidney Kimmel Comprehensive Cancer Center, Johns Hopkins Medicine, Baltimore, MD USA; 6 Division of Cancer Epidemiology & Genetics, NIH-NCI Clinical Genetics Branch, Rockville, MD USA; 7 Clinical Research Division, Fred Hutchinson Cancer Research Center, Seattle, WA USA; 8 Cleveland Clinic Foundation, Cleveland, OH USA; 9 Stem Cell Transplantation and Cellular Therapy, Dana-Farber Cancer Institute, Boston, MA USA; 10 Department of Medicine, Baylor College of Medicine, Houston, TX USA; 11 Division of Biostatistics, Institute for Health & Equity, and Cancer Center, Medical College of Wisconsin, Milwaukee, WI USA; 12 Division of Hematology and Oncology, Department of Medicine, CIBMTR® (Center for International Blood and Marrow Transplant Research), Medical College of Wisconsin, 9200 W Wisconsin Ave, Milwaukee, WI 53226 USA; 13 Cancer Center Biostatistics Shared Resource, Medical College of Wisconsin, Milwaukee, WI USA

**Keywords:** Mitochondrial genome, Allogeneic hematopoietic stem-cell transplantation, Myelodysplastic syndromes, Prognosis, Whole-genome sequencing

## Abstract

Despite mitochondrial DNA (mtDNA) mutations are common events in cancer, their global frequency and clinical impact have not been comprehensively characterized in patients with myelodysplastic neoplasia (also known as myelodysplastic syndromes, MDS). Here we performed whole-genome sequencing (WGS) on samples obtained before allogenic hematopoietic cell transplantation (allo-HCT) from 494 patients with MDS who were enrolled in the Center for International Blood and Marrow Transplant Research. We evaluated the impact of mtDNA mutations on transplantation outcomes, including overall survival (OS), relapse, relapse-free survival (RFS), and transplant-related mortality (TRM). A random survival forest algorithm was applied to evaluate the prognostic performance of models that include mtDNA mutations alone and combined with MDS- and HCT-related clinical factors. A total of 2666 mtDNA mutations were identified, including 411 potential pathogenic variants. We found that overall, an increased number of mtDNA mutations was associated with inferior transplantation outcomes. Mutations in several frequently mutated mtDNA genes (*e.g*., *MT-CYB* and *MT-ND5*) were identified as independent predictors of OS, RFS, relapse and/or TRM after allo-HCT. Integration of mtDNA mutations into the models based on the Revised International Prognostic Scores (IPSS-R) and clinical factors related to MDS and allo-HCT could capture more prognostic information and significantly improve the prognostic stratification efforts. Our study represents the first WGS effort in MDS receiving allo-HCT and shows that there may be clinical utility of mtDNA variants to predict allo-HCT outcomes in combination with more standard clinical parameters.

**Supplementary Information:**

The online version contains supplementary material available at 10.1186/s13045-023-01418-4.

**To the Editor,**

Myelodysplastic neoplasia (also known as myelodysplastic syndromes, MDS) are a heterogenous group of clonal hematopoietic cell disorders characterized by blood cytopenias and a tendency to progress to acute myeloid leukemia (AML) [1]. Despite treatment advances, allogeneic hematopoietic stem-cell transplantation (allo-HCT) remains the only potentially curative therapy for MDS. However, mortality after allo-HCT is high due to disease relapse and transplant-related complications [1]. Deciding which MDS patients will most likely benefit from allo-HCT is challenging given the clinical and biological heterogeneity of the disease [1]. Previous genomic analyses have shown that mutations in specific nuclear genes (*e.g*., *TP53*) could inform prognostic stratification of MDS undergoing allo-HCT [2]. Surprisingly, despite the critical role of mitochondria in energy production, heme biosynthesis and metabolism [3], there has not yet been a comprehensive evaluation of mutations in the mitochondrial genome on transplant outcomes for MDS.

To address this knowledge gap, we performed whole-genome sequencing (WGS) on whole blood samples obtained before allo-HCT from 494 patients with MDS and analyzed their mitochondrial genomes (Additional file 2: Table S1). All patients were of European ancestry. Details of mtDNA analysis are provided in Additional file 3. We identified 2666 mtDNA variants (2542 substitutions and 124 small indels), 250 (9.4%) of which have not been reported in the MITOMAP database (Fig. 1A). Among them, 411 variants are putative pathogenic, with the majority (95%) having low allele frequency (AF < 1%) in our cohort. This is consistent with the ACMG/AMP assessment that pathogenic variants tend to be rare [4]. The mitochondrial control region (also known as the D-Loop) was the most frequently mutated region with 14.4% of the variants located in this region (Fig. 1B and C). The most frequently mutated protein-coding gene was *MT-ND5* (16%), followed by *MT-CO1* (14%). Eight percent of the variants were in the mitochondrial *MT-tRNA* genes. Mutational signature analysis showed that T > C and C > T transitions were the predominant substitutions in MDS (Additional file 1: Fig. S1). These results are consistent with previous reports from small-scale MDS studies [5].

**Fig. 1:**
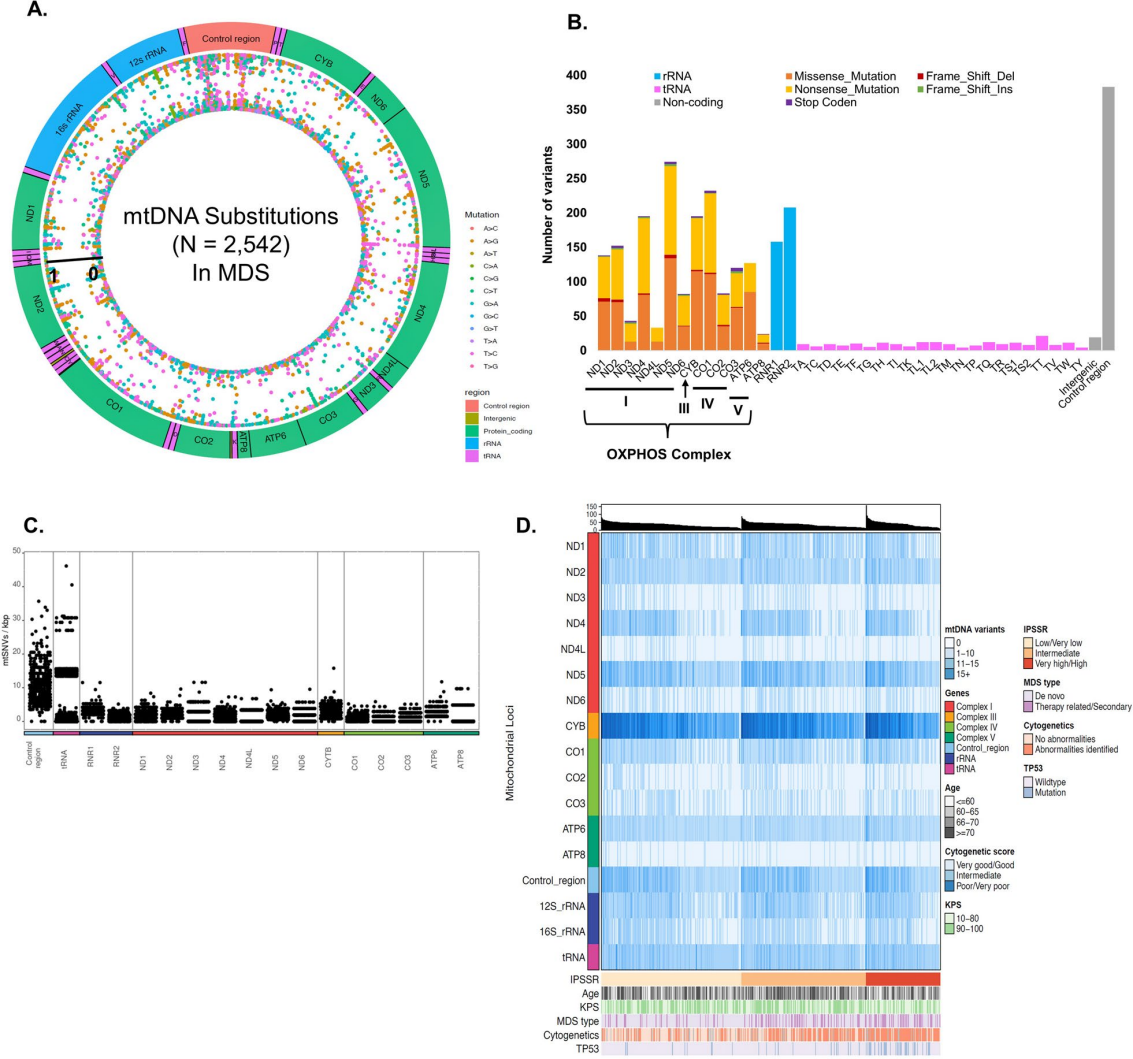
Mitochondrial genomic landscape in MDS. **A** Human mtDNA map showing genes and intergenic regions in the circular plot. Colors of the circular bars represent mtDNA regions. mtDNA encodes 22 transfer RNAs (tRNAs), two ribosomal RNAs (rRNAs), and 13 proteins for the mitochondrial OXPHOS apparatus. These 13 components include seven (*ND1, 2, 3, 4, 4L, 5, 6*) of complex I, one (*CYB*) of the 11 subunits of complex III, three (*CO1, 2, 3*) of the 13 subunits of complex IV, and two (*ATP6, 8*) of the 16 subunits of complex V. Dots inside the circular plot represent substitutions of mtDNA identified in 494 MDS patients. Classes of substitutions are presented color-coded. Vertical axes represent the heteroplasmic fraction of each variant. **B** Number of mtDNA variants at different mtDNA genes. Colors represent the biotype of the variant. **C** Frequency and distribution of variants within the mitochondrial genome. Variant frequency normalized by dividing the number of variants per locus of each patient by length of the locus (kbp). **D** Associations between mtDNA variants and MDS clinical factors. The top panel displays the number of mtDNA variants per patient sorted first by the IPSS-R score, then by the number of mtDNA variants. A heatmap showing the location of each variant on the mitochondrial genome (middle), where the color of each bar represents the number of mtDNA variants in each gene. The mitochondrial genes are represented on the left. The bottom panel shows the clinical covariates for all 494 patients: Age, IPSS-R score, KPS, MDS types and present of cytogenetic abnormities and *TP53* mutational status

The median number of mtDNA mutations identified per patient was 37 (ranging from 4 to 157). Presence of mtDNA mutations was not associated with patient age at transplant, Karnofsky Performance Score (KPS), the Revised International Prognostic Score (IPSS-R), MDS types, or cytogenetic abnormalities (all *P* > 0.05, Fig. 1D). Overall, an increase in the number of mitochondrial mutations was significantly associated with a decrease in overall survival (OS) (HR, 1.11; 95% CI, 1.01–1.22; *P* = 0.029), higher risk of relapse (HR, 1.13; 95% CI, 1.00–1.27; *P* = 0.049) and shorter relapse-free survival (RFS) (HR, 1.13; 95% CI, 1.04–1.24; *P* = 0.007). Analyses for each putative pathogenic variant observed 13 rare variants (11 missense and 2 stop-gain) that were associated with at least one of the four post-transplant outcomes (*i.e.,* OS, relapse, RFS, and transplant-related mortality (TRM)) after Bonferroni correction (*P* < 0.05/411 = 1.22 × 10^–4^) (Additional file 2: Table S2). All these 13 variants were present in a heteroplasmic state, with HF levels ranging from 1% to 68.2%. Common variants (AF ≥ 1%) that were associated with posttransplant outcomes at *P* values < 0.05 were listed in Additional file 2: Tables S3–S6, 23 of which were associated with more than one outcome. Among them, *MT-CO3* m.9656 T > C, *MT-ND2* m.5495 T > C, and *MT-CYB* m.15607A > G reached Bonferroni-adjusted threshold (*P* < 0.05/27 = 1.85 × 10^–3^) in the conditional analysis. The Fine-Gray model derived similar results for relapse and TRM, with minor differences (Additional file 2: Tables S5–S6).

Gene-based analyses yielded significant associations with OS for *MT-CYB* (*P* = 1.04 × 10^–3^), *MT-ND2* (*P* = 3.06 × 10^–3^) and *MT-ND4* (*P* = 1.41 × 10^–3^) after Bonferroni correction (*P* < 0.05/16 = 3.13 × 10^–3^). For RFS, significant associations were observed for *MT-CYB* (*P* = 2.78 × 10^–3^) and *MT-ND4* (*P* = 7.75 × 10^–4^). Four mtDNA genes were significantly associated with relapse, including *MT-CYB* (*P* = 7.03 × 10^–4^), *MT-ND2* (*P* = 5.92 × 10^–4^), *MT-ND5* (*P* = 1.91 × 10^–3^) and *MT-tRNA* (*P* = 1.99 × 10^–4^). Two mtDNA genes (*MT-CYB* and *MT-ND4L*) were significantly associated with TRM, with *P* values of 2.24 × 10^–3^ and 4.91 × 10^–4^ for *MT-CYB* and *MT-ND4L*, respectively (Additional file 2: Table S7). Additional significantly associated mitochondrial genes were also observed in burden test and/or SKAT (Additional file 2: Table S8 and Additional file 1: Figs. S2–S5). Most of these associated genes are located on the mitochondrial electron transport chain (ETC). ETC function is coupled to oxidative phosphorylation and the production of metabolites by the tricarboxylic acid cycle [6]. Mutations on these genes could result in complex dysfunction and abnormal reactive oxygen species production, which further promotes tumorigenesis and tumor progression [7].

Sixteen haplogroups were predicted in our cohort. Consistent with previous studies in non-Hispanic whites, haplogroup H was the most common haplogroup in our cohort [8]. Compared to haplogroup H, haplogroup I was significantly associated with worse OS (HR, 2.32; 95% CI, 1.23–4.35; *P* = 0.01) and shorter RFS (HR, 2.04; 95% CI, 1.09–3.83; *P* = 0.03). Haplogroup K was significantly associated with increased risk of relapse (HR, 1.70; 95% CI, 1.03–2.81; *P* = 0.04) (Additional file 2: Table S9).

To investigate whether mtDNA mutations could improve the prognostic stratification of MDS receiving allo-HCT, we fitted random survival forest models with and without inclusion of mtDNA mutations in the models (Additional file 1: Fig. S6). The model based only on mtDNA genes had a c-index of 0.58 to predict OS, which was slightly higher than the IPSS-R (c-index = 0.48) and clinical model (IPSS-R plus MDS type and pre-transplantation treatments, c-index = 0.57). Adding mtDNA genes improved the predictive performance of the model, with the c-index increasing from 0.48 to 0.63 for the IPSS-R model and from 0.57 to 0.66 for the clinical model (Fig. 2). Similar results were observed for the models in predicting RFS, relapse and TRM. Because IPSS-R is the currently available clinical scoring system to predict OS and leukemic transformation of MDS, we further examined the reclassification properties of adding mtDNA mutations to the IPSS-R model in predicting OS and relapse (Additional file 1: Fig. S7). The model with mtDNA mutations up-staged 18.6%, 41.7%, and 52.5% of the patients having lower-risk IPSS-R scores and down-staged 35.1%, 14.7% and 1.6% of the patients having higher-risk IPSS-R scores for 1-, 2-, and 5-year OS, respectively. For relapse, the model with mtDNA mutations up-staged 18.8%, 22.0%, and 22.0% of the patients having lower-risk IPSS-R scores and down-staged 26.2%, 35.6% and 35.6% of the patients having higher-risk IPSS-R scores for 1-, 2-, and 5-year risk of relapse, respectively.

**Fig. 2:**
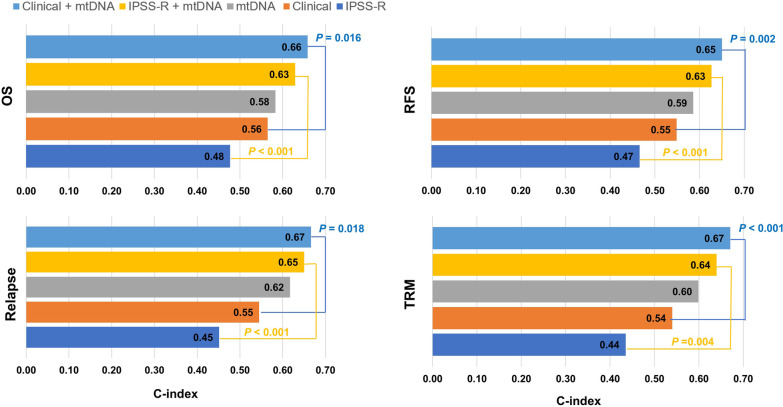
Prognostic impact of mtDNA variants in MDS. Prognostic models are presented color-coded. X-axes represent the C-index of the each model, with the values of the C-index showing inside of each bar. *P* values are the differences between the models with and without mtDNA variants

Most recently, Bernard et al*.* developed a clinical-molecular prognostic model, termed the IPSS-Molecular (IPSS-M) model, which combines somatic mutations of 31 genes with hematologic and cytogenetic parameters [9]. The IPSS-M improved prognostic discrimination across all clinical end points and reclassified 46% of MDS patients as compared to the IPSS-R model, demonstrating the importance and clinical utility of recurrent mutations in MDS risk stratification [9]. Although our current analysis focused on the prognostic significance of mtDNA mutations, to investigate whether mtDNA mutations could provide additional prognostic stratification to the recurrent mutations, we further conducted stratified analysis by *TP53* mutation status. We chose *TP53* mutation as an example because our group and others have repeatedly identified *TP53* mutation as a powerful predictor of MDS survival after transplantation [2, 9–11]. However, more than 80% of MDS patients do not carry *TP53* mutations. In these patients, additional prognostic markers are needed to further stratify their posttransplant outcomes [2, 10, 11]. In our MDS cohort, *TP53* mutations were present in 11% of the patients and as expected, were associated with shorter OS, increased risk of relapse, shorter RFS and worse TRM than those without *TP53* mutations (all log-rank *P* < 0.01, Additional file 1: Fig. S8). The presence of *TP53* mutations was not correlated with the number of mtDNA mutations, nor the mutation status of each mtDNA gene (all *P* > 0.05, Fig. 1D). Of note, mtDNA mutations could provide additional prognostic stratification for patients who don’t carry *TP53* mutations and improved the predictive performance of the models based on the IPSS-R and clinical factors (Additional file 2: Table S10). These findings suggest that mtDNA mutations could provide additional prognostic stratification information to the recurrent nuclear DNA mutations. However, we did not systematically evaluate the accumulated effects of nuclear DNA mutations and mtDNA mutations on patient survival, which requires further investigation.

In conclusion, this was the first attempt to characterize mtDNA genomic landscape in MDS receiving allo-HCT. Our results provide novel insights into the clinical utility of mtDNA mutations and could serve as a proof of concept that integration of mtDNA mutations into the scoring system to improve the clinical decision-making. Further studies are needed to better understand the biological mechanisms, and new techniques for mitochondrial genome editing are required to help restore mitochondrial function and develop mitochondria-targeted therapy.

## Supplementary Information

**Additional file 1.** Supplementary Figures.

**Additional file 2.** Supplementary Tables.

**Additional file 3.** Supplementary Methods.

## Data Availability

CIBMTR supports accessibility of research in accord with the National Institutes of Health (NIH) Data Sharing Policy and the National Cancer Institute (NCI) Cancer Moonshot Public Access and Data Sharing Policy. The CIBMTR only releases de-identified datasets that comply with all relevant global regulations regarding privacy and confidentiality.

## Abbreviations

allo-HCT: Allogeneic hematopoietic stem-cell transplantation
MDS: Myelodysplastic syndromes
mtDNA: Mitochondrial DNA
WGS: Whole-genome sequencing
CIBMTR: Center for International Blood and Marrow Transplant Research
OS: Overall survival
RFS: Relapse-free survival
TRM: Transplant-related mortality
KPS: Karnofsky Performance Score
IPSS: International Prognostic Scoring System
IPSS-R: Revised International Prognostic Scoring System
ROS: Reactive oxygen species
OXPHOS: Oxidative phosphorylation
tRNA: Transfer RNA
rRNA: Ribosomal RNA
ETC: Electron transport chain
HF: Heteroplasmic fraction
AF: Allele frequency
SKAT-O: Sequence kernel association test
