# Supplementary material for: Prognostic landscape of mitochondrial genome in myelodysplastic syndrome after stem-cell transplantation
Source: J Hematol Oncol. 2023 Mar 10;16:21. doi: 10.1186/s13045-023-01418-4 (PMC9999628; doi:10.1186/s13045-023-01418-4)

**Supplementary Figure 1. Mitochondrial genome mutational signatures in MDS.** (a) Single base substitutions (SBS); (b) Doublet base substitutions (DBS); (c) Small insertions and deletions (ID).


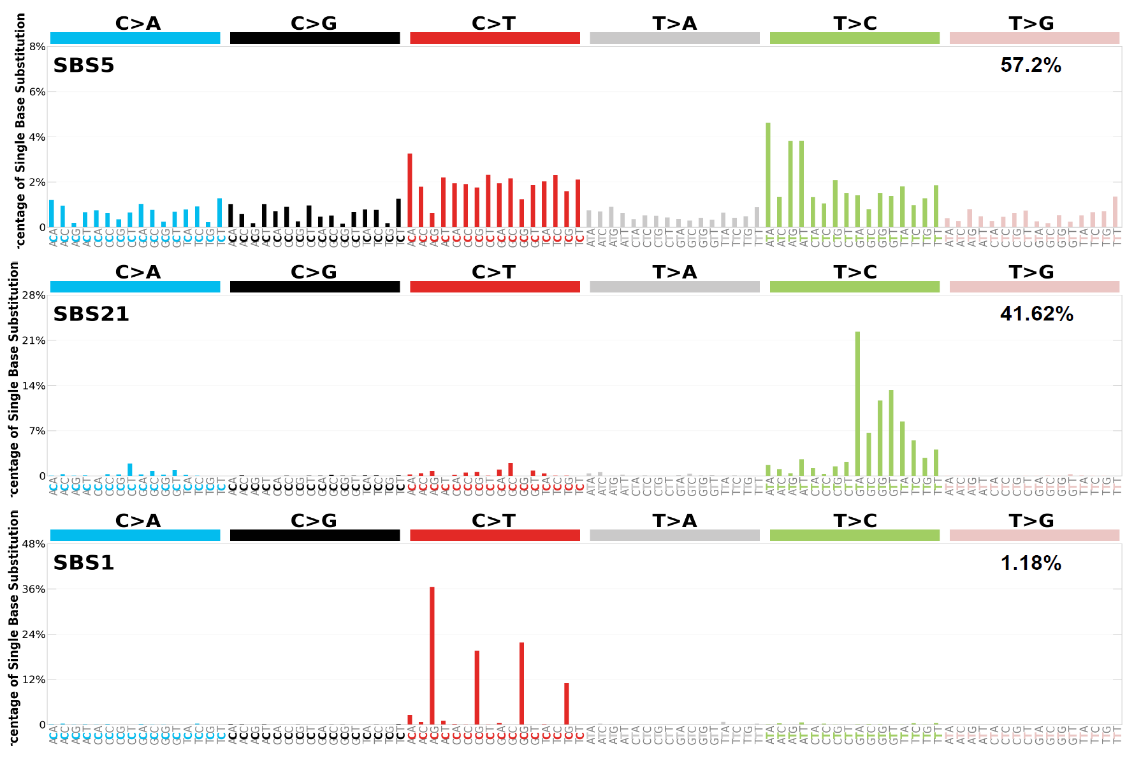


**a**

**b**


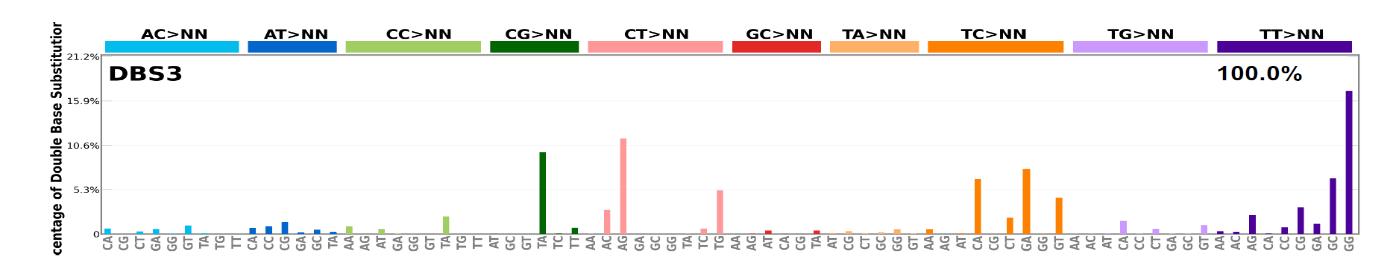


**
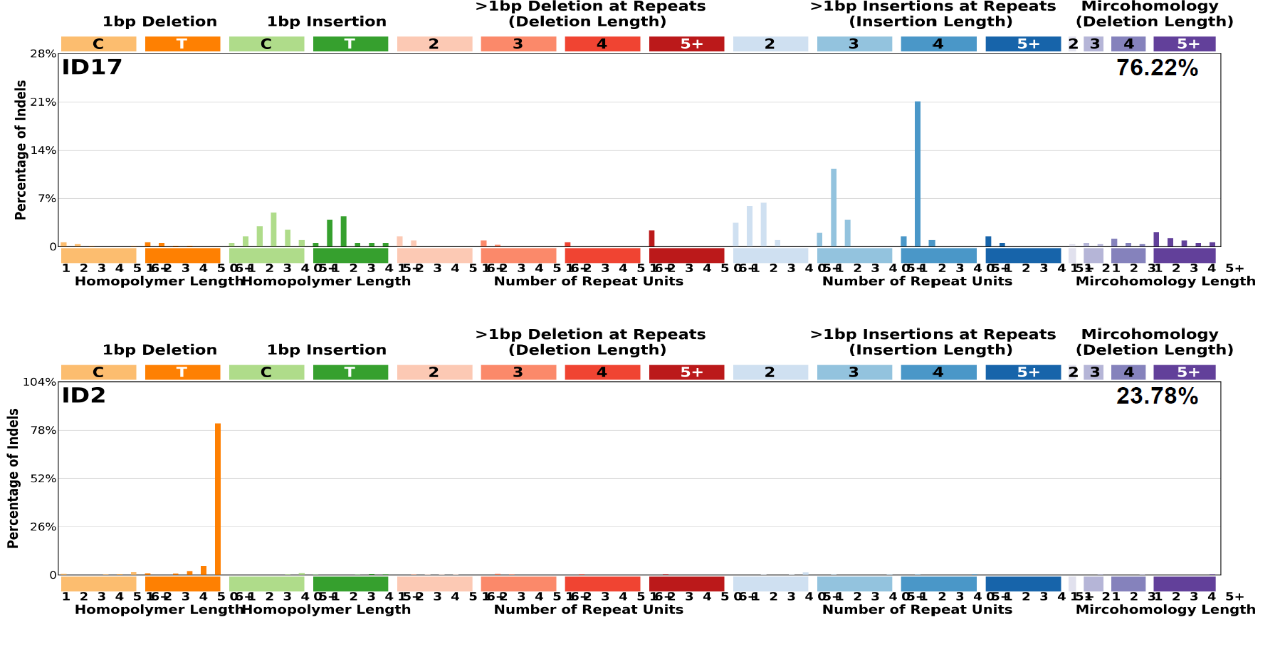
**

**c**

**Supplementary Figure 2. Survival Curves for OS based on mtDNA gene variants.**


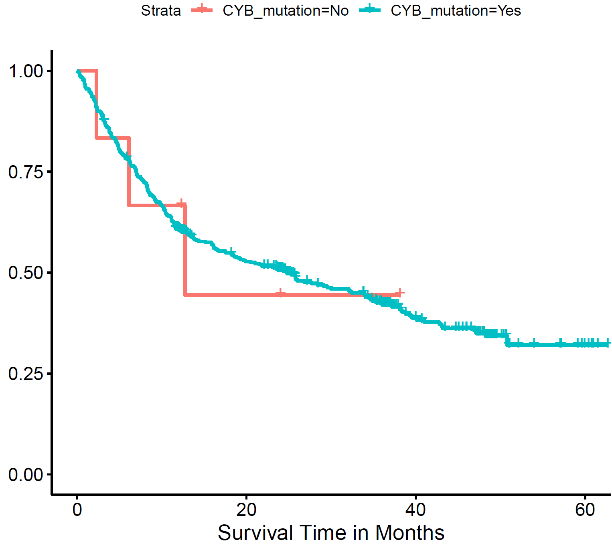

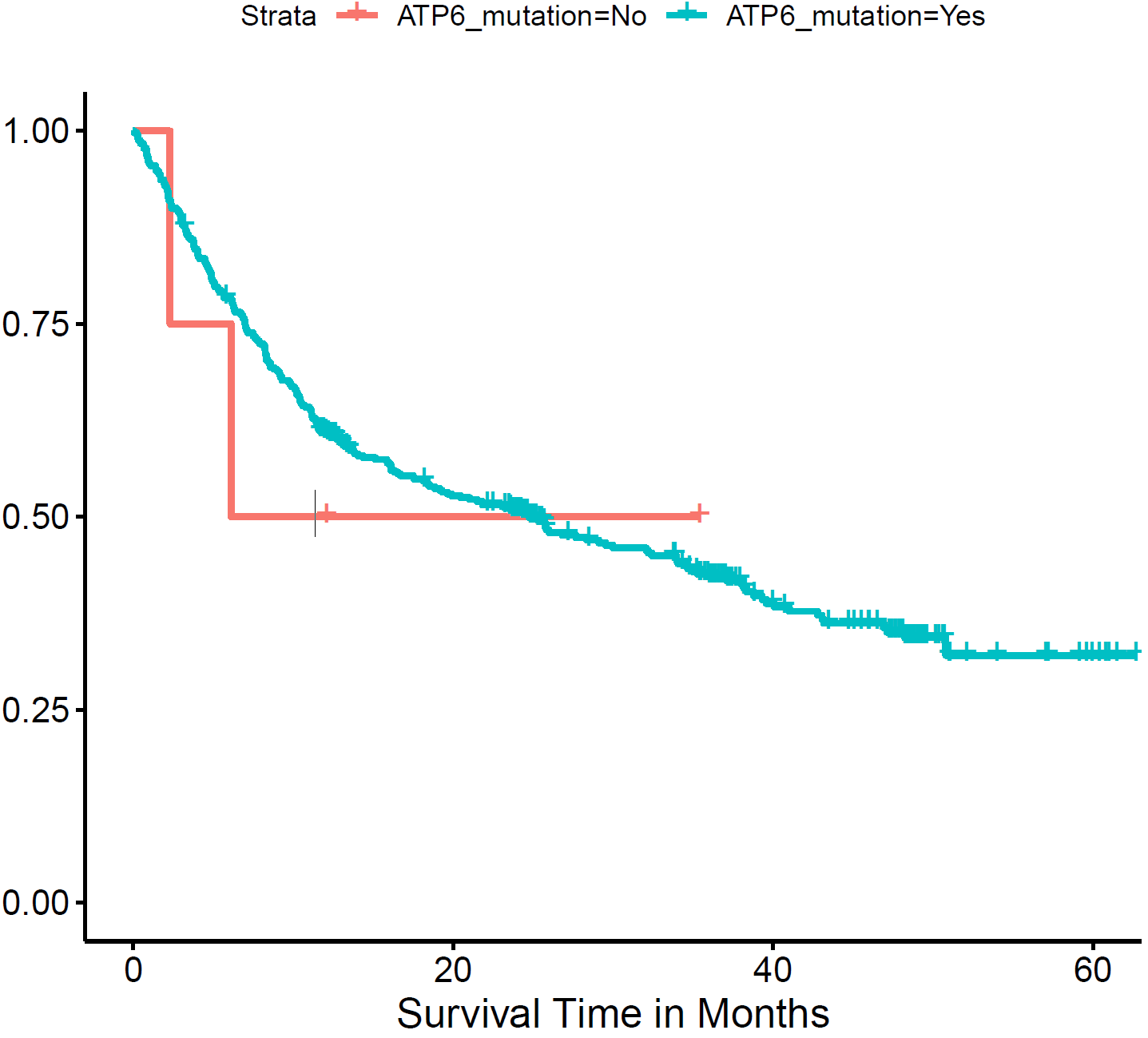

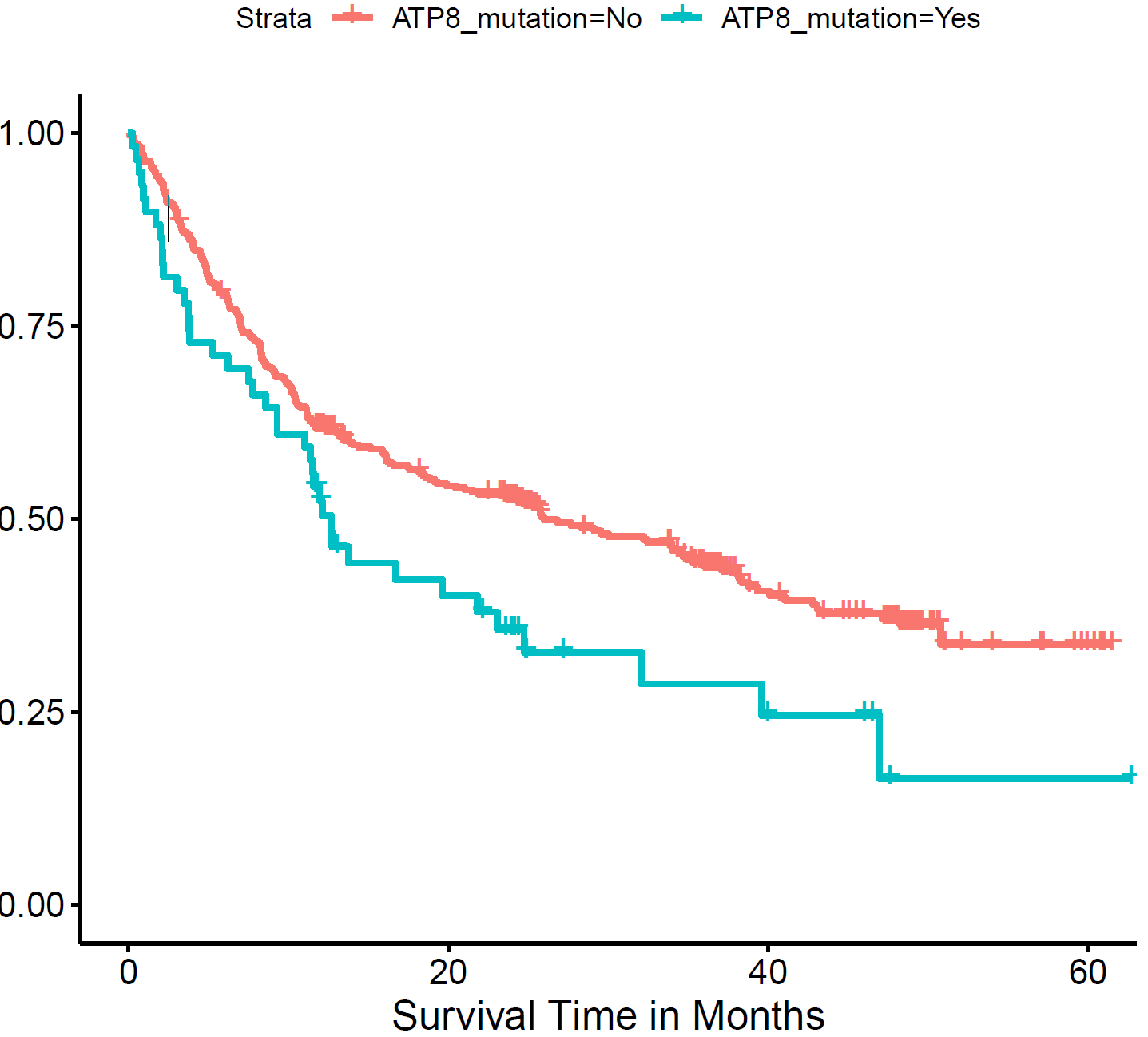


Survival probability

*CYB*

Log-Rank *P* = 0.90

*ATP8*

Log-Rank *P* = 0.01

*ATP6*

Log-Rank *P* = 0.81


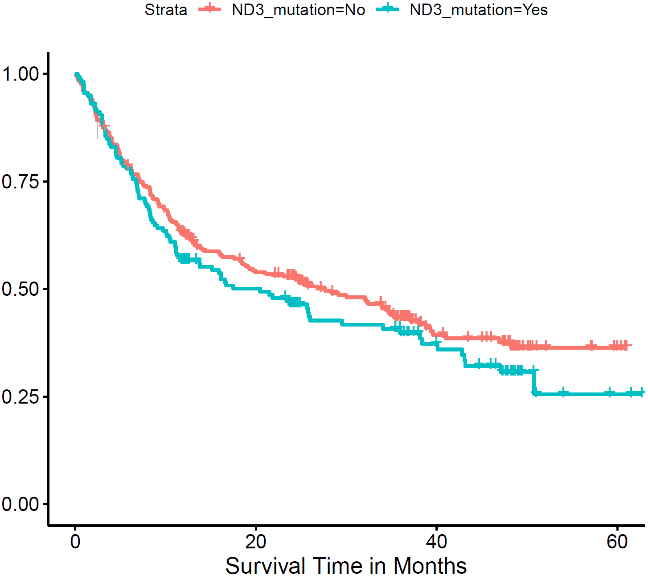

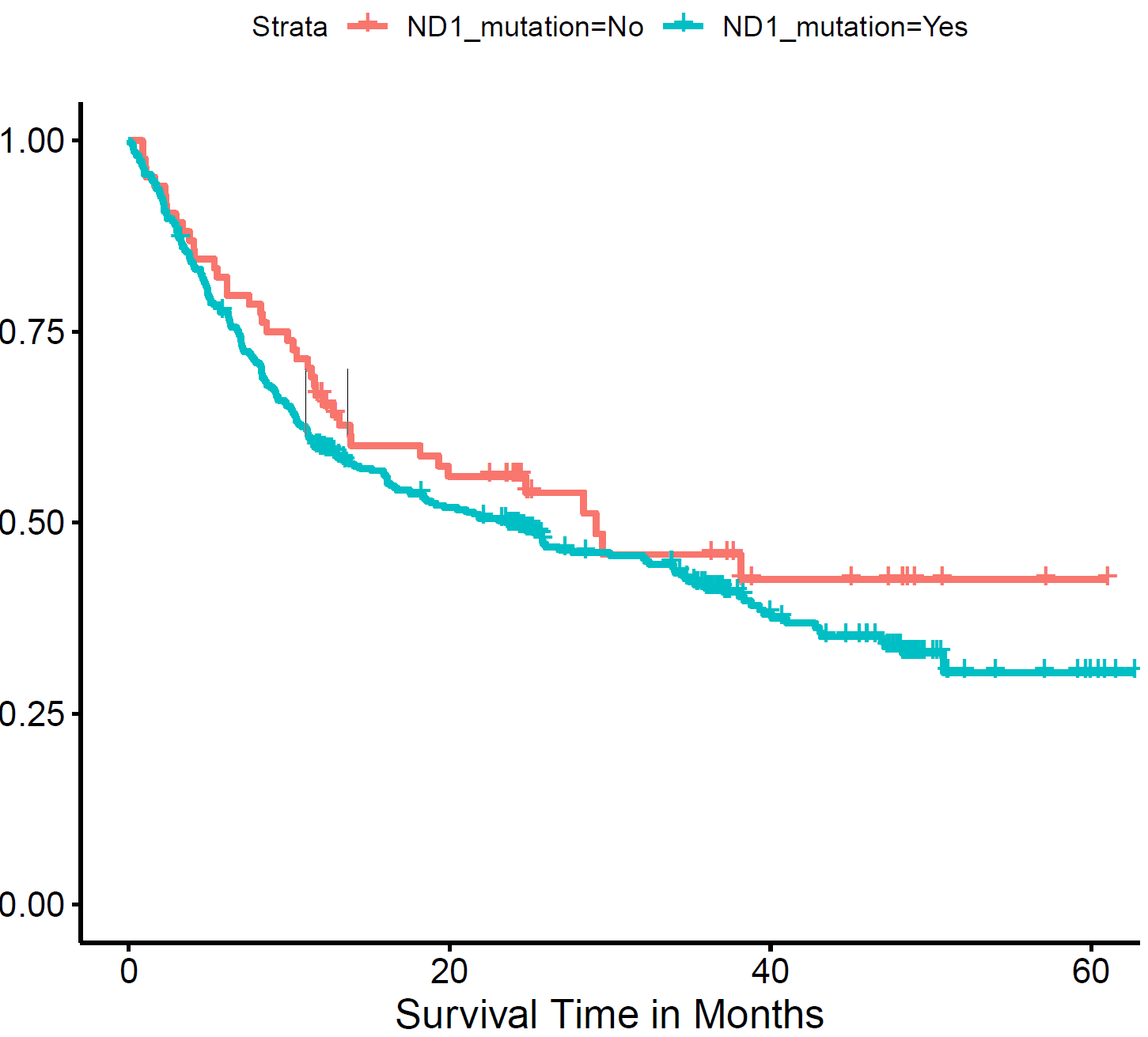

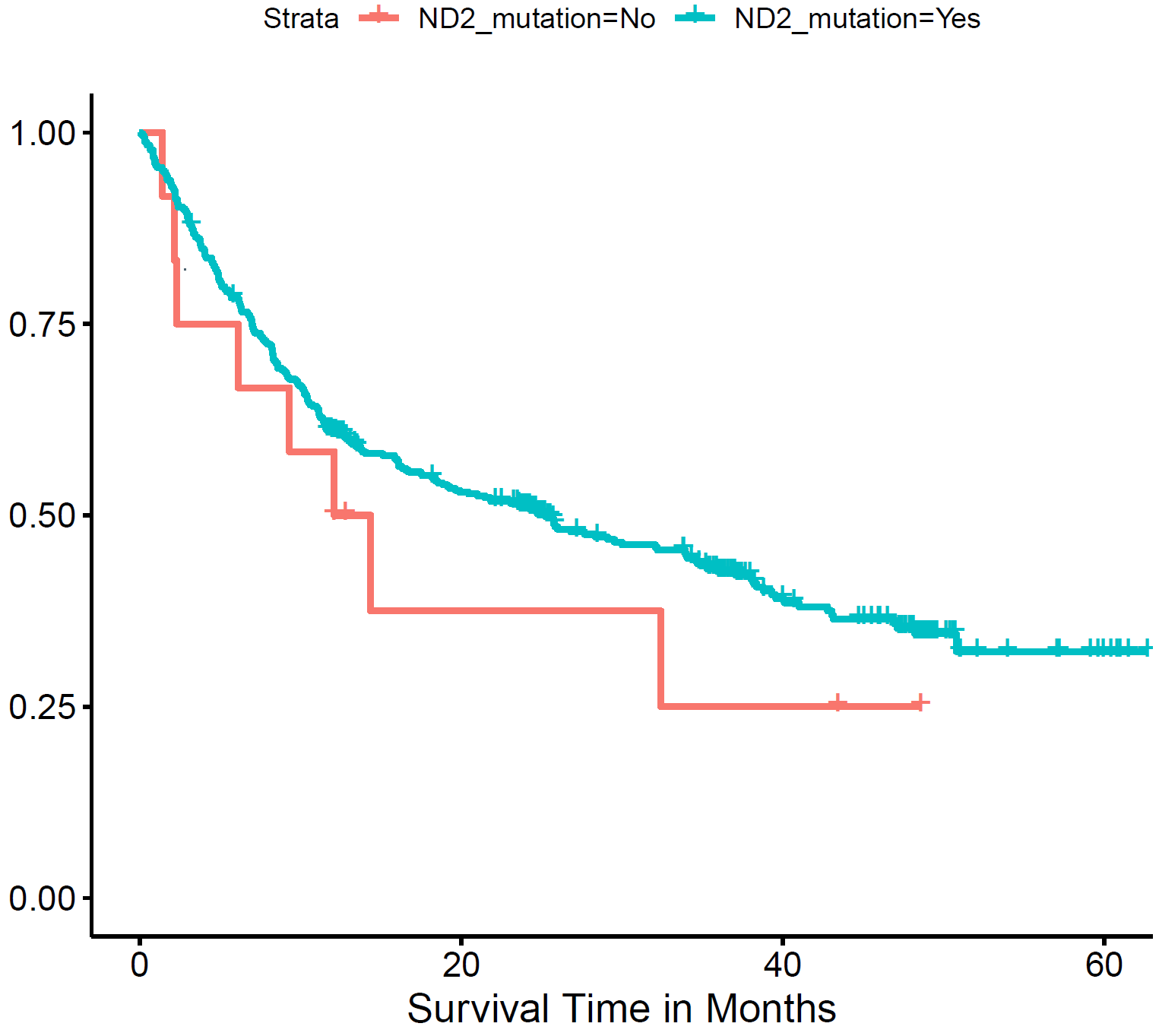


*ND2*

Log-Rank *P* = 0.36

*ND1*

Log-Rank *P* = 0.23

*ND3*

Log-Rank *P* = 0.21

Survival probability


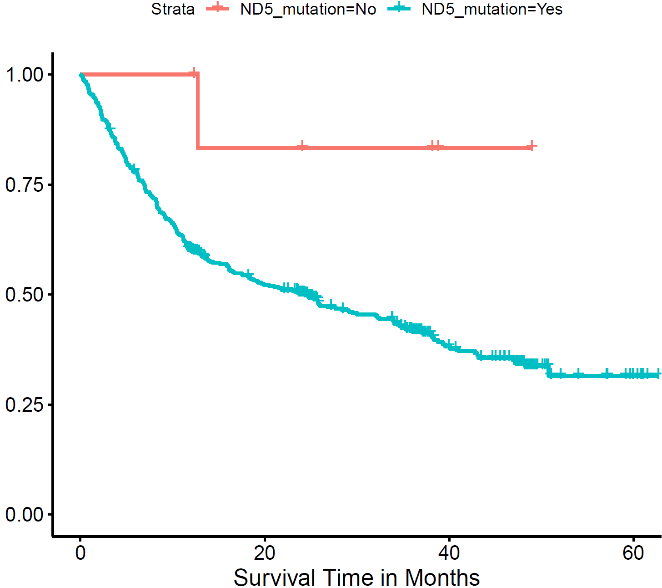

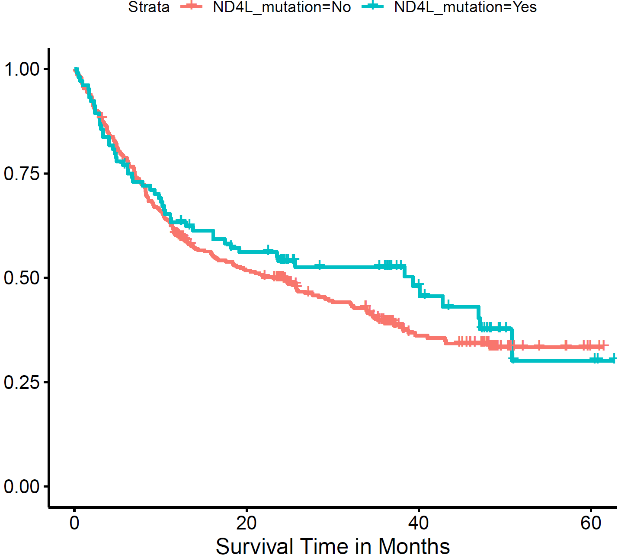

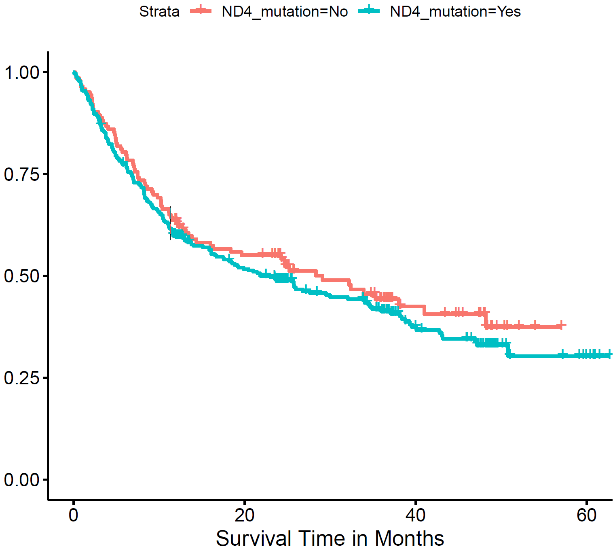


*ND5*

Log-Rank *P* = 0.06

*ND4L*

Log-Rank *P* = 0.33

*ND4*

Log-Rank *P* = 0.36

Survival probability


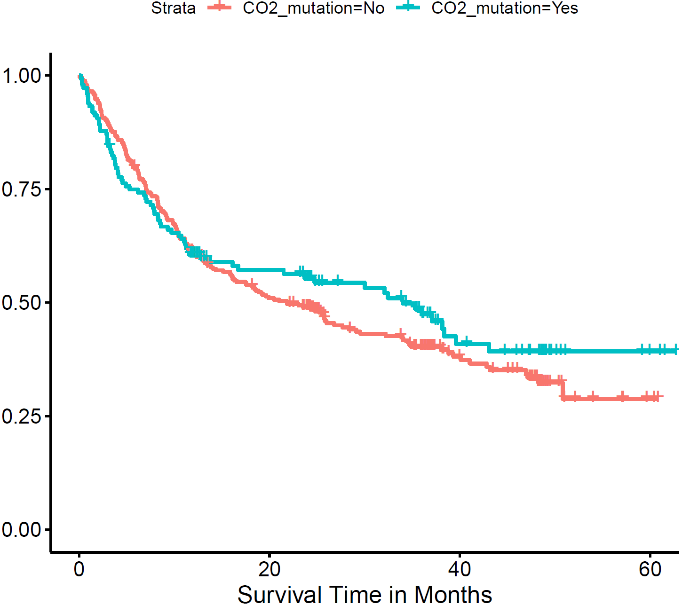

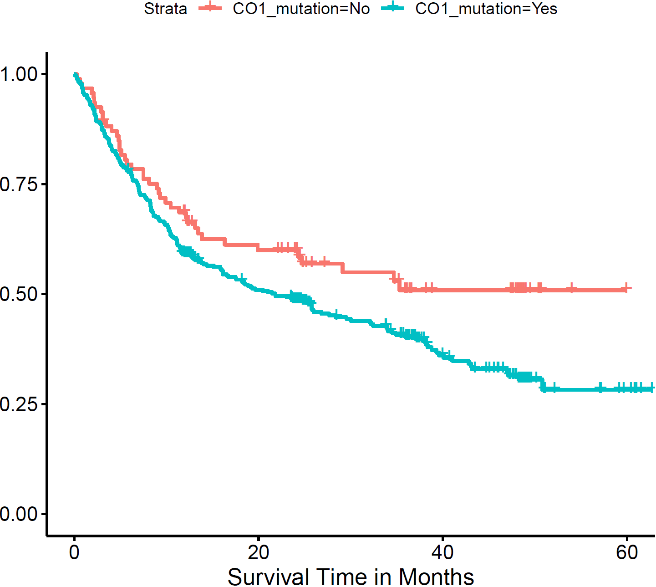

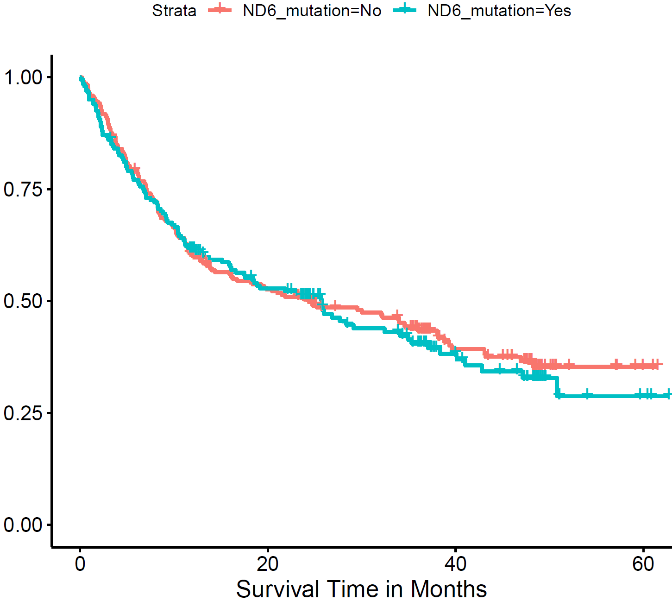


*CO2*

Log-Rank *P* = 0.42

*CO1*

Log-Rank *P* = 0.03

*ND6*

Log-Rank *P* = 0.72

Survival probability

Survival probability


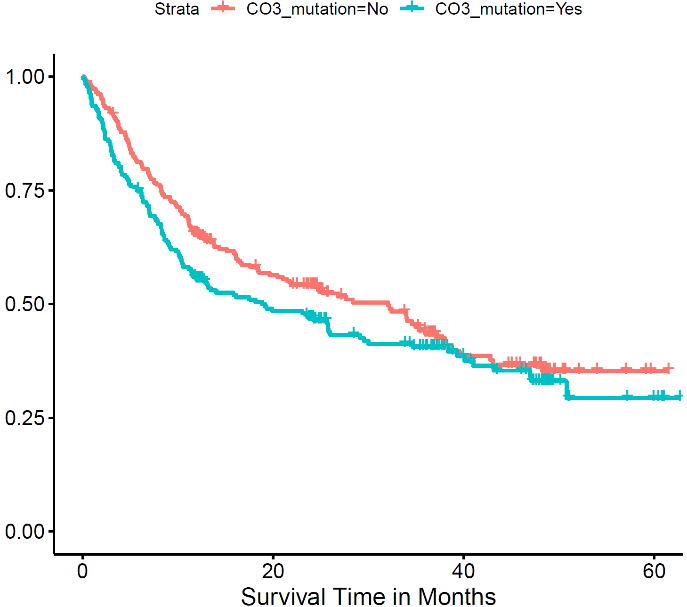

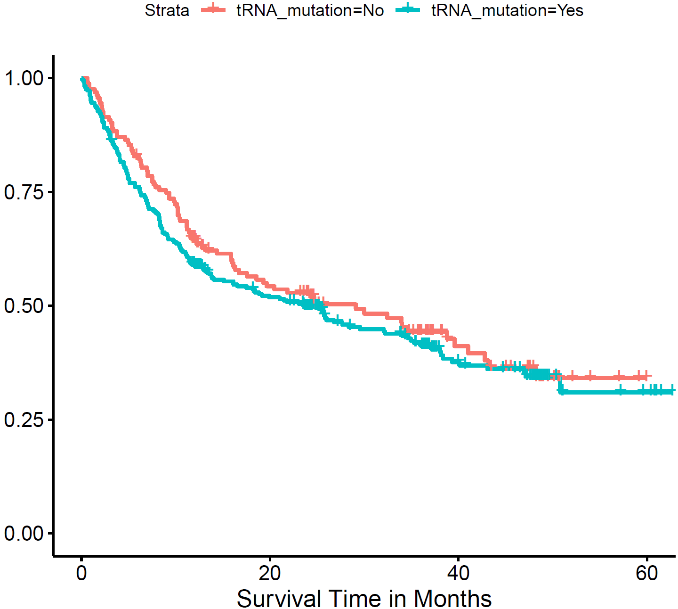

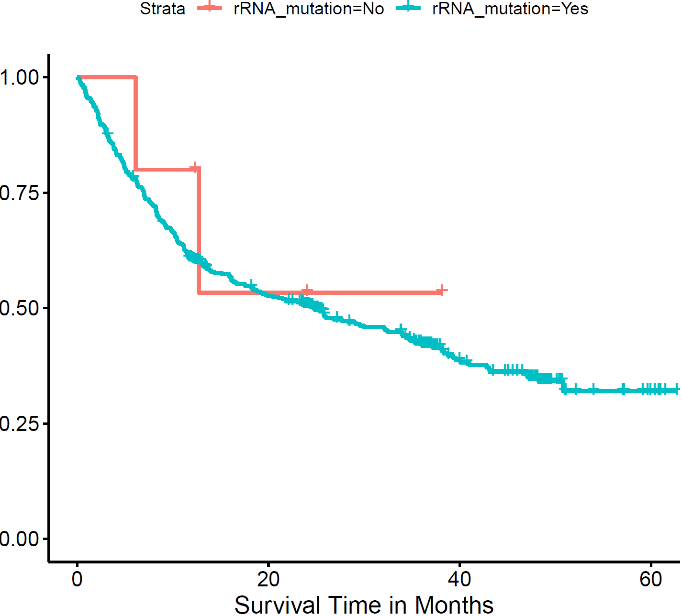


*rRNA*

Log-Rank *P* = 0.62

*CO3*

Log-Rank *P* = 0.10

*tRNA*

Log-Rank *P* = 0.39

**Supplementary Figure 3. Survival Curves for RFS based on mtDNA gene variants.**

Survival probability


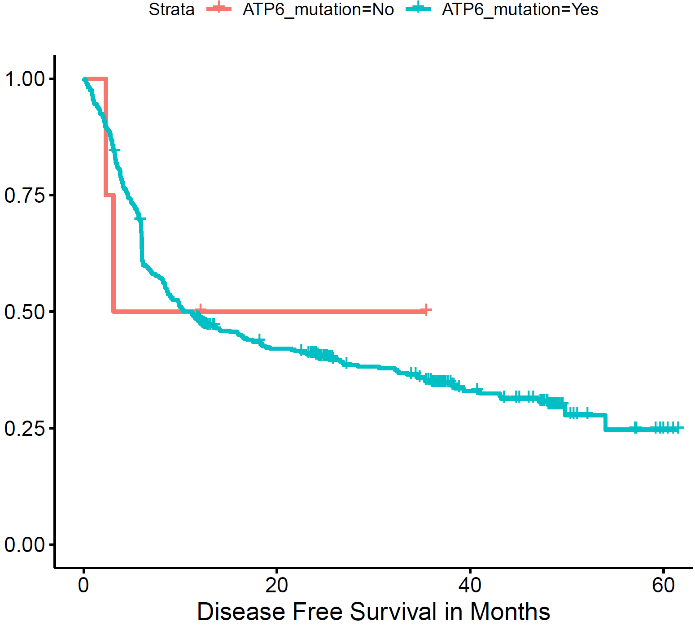

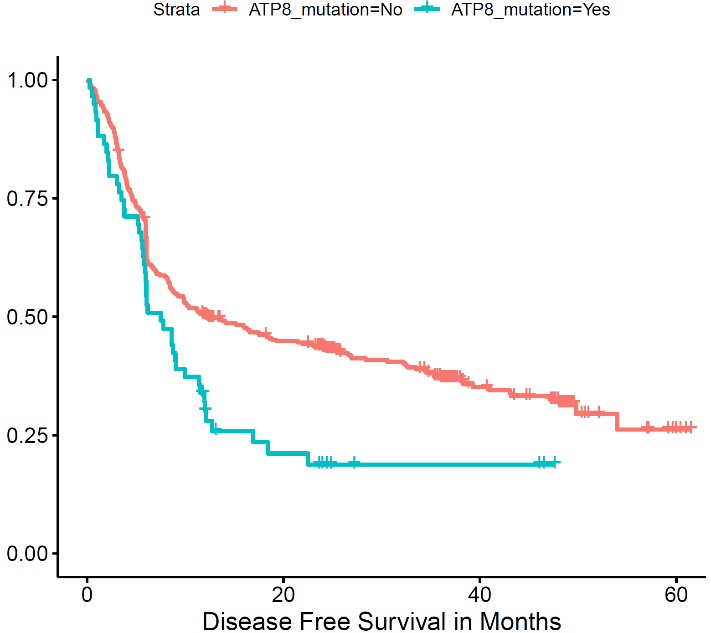

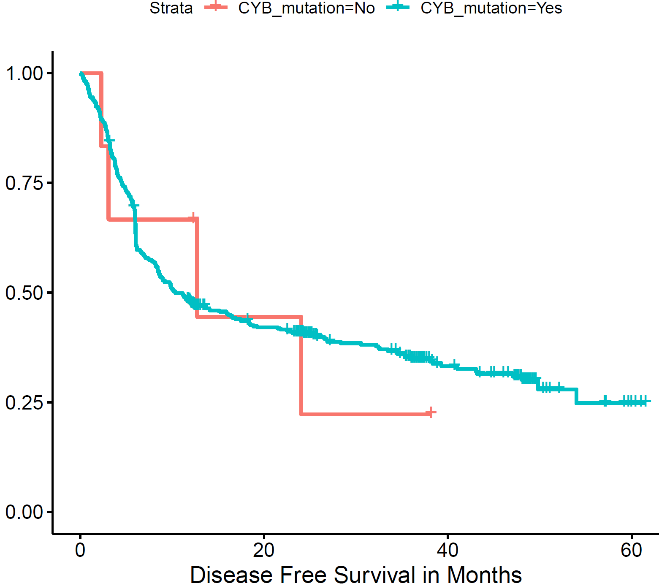


*ATP6*

Log-Rank *P* = 0.96

*ATP8*

Log-Rank *P* = 0.002

*CYB*

Log-Rank *P* = 0.89


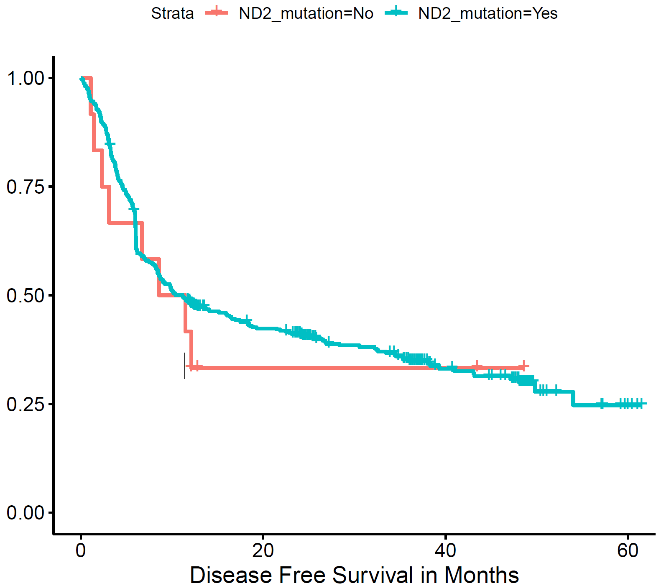

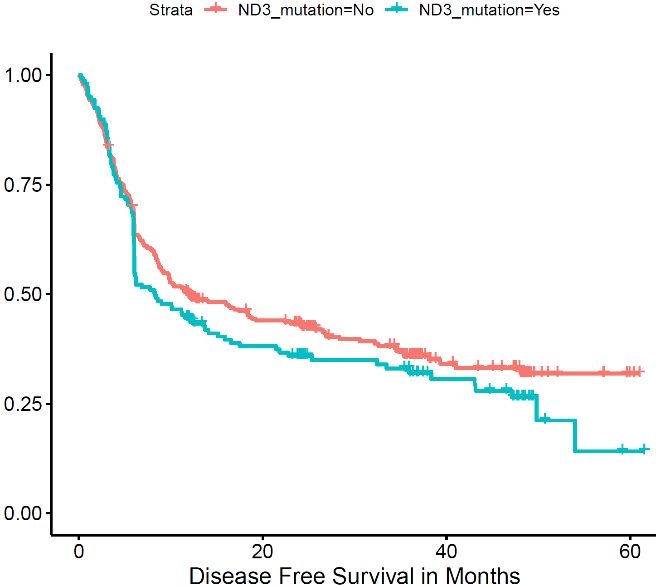

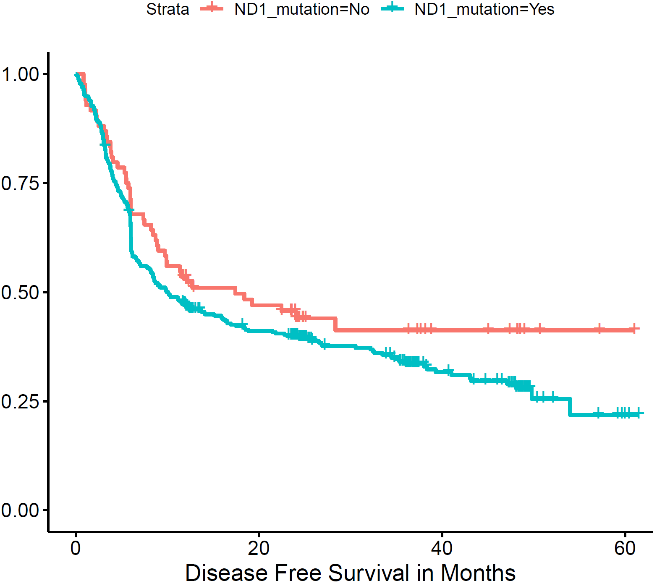


Survival probability

*ND2*

Log-Rank *P* = 0.65

*ND3*

Log-Rank *P* = 0.15

*ND1*

Log-Rank *P* = 0.15


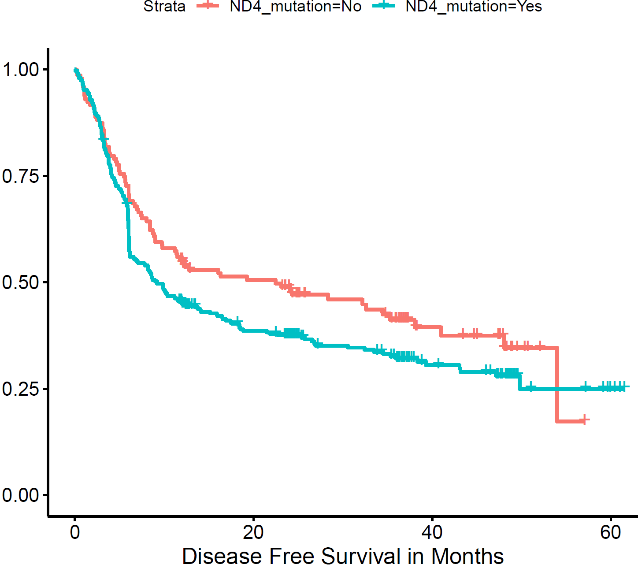


*ND4*

Log-Rank *P* = 0.05


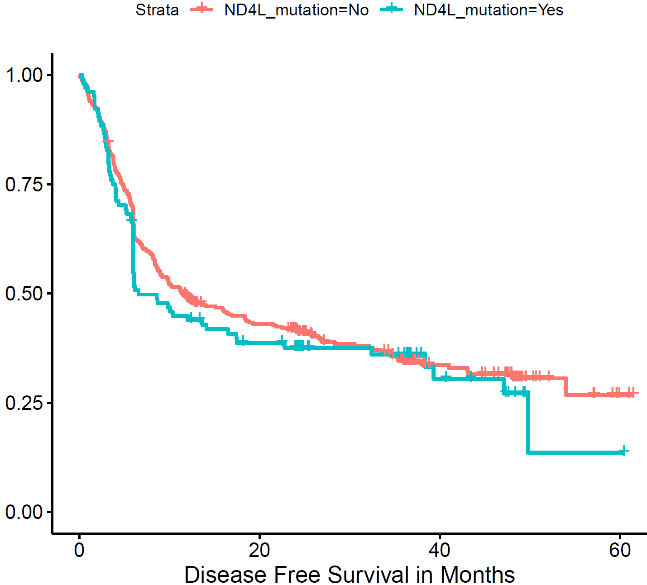


*ND4L*

Log-Rank *P* = 0.36


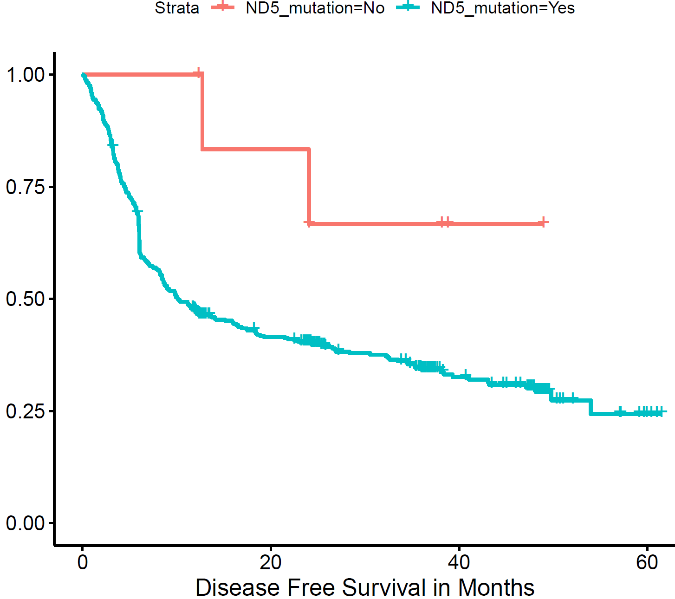


*ND5*

Log-Rank *P* = 0.07

Survival probability

Survival probability


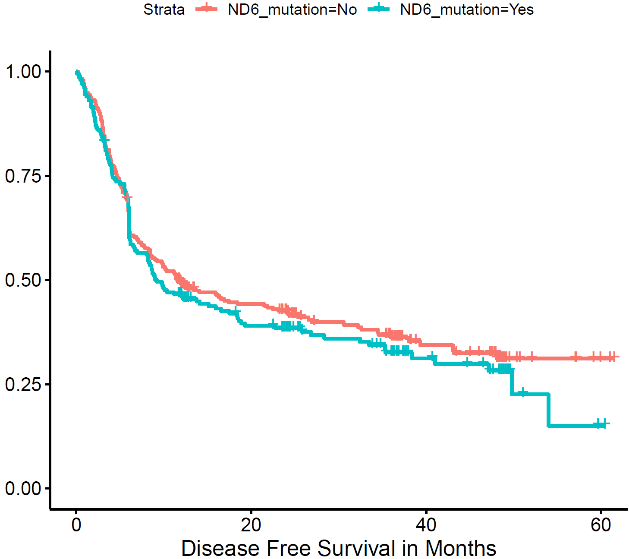

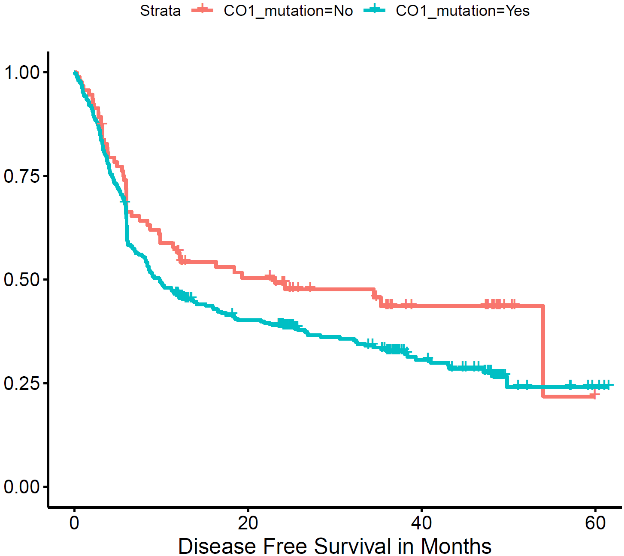

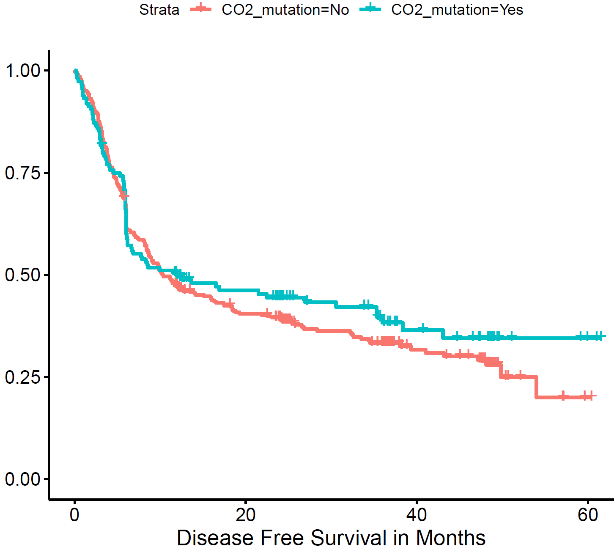


*ND6*

Log-Rank *P* = 0.35

*CO1*

Log-Rank *P* = 0.04

*CO2*

Log-Rank *P* = 0.41


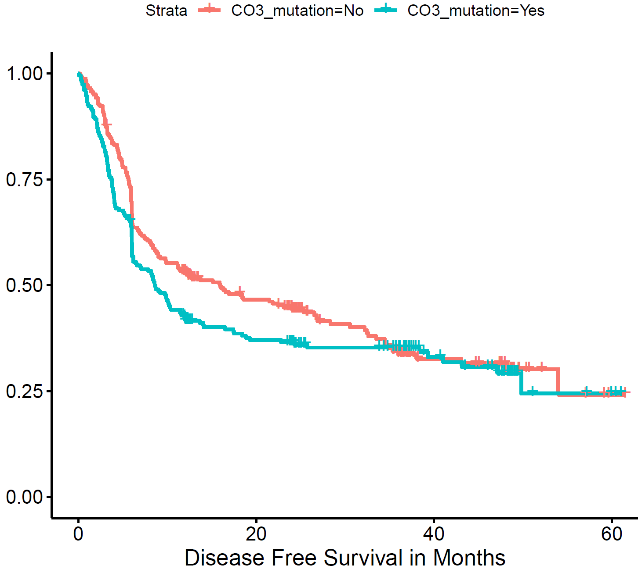

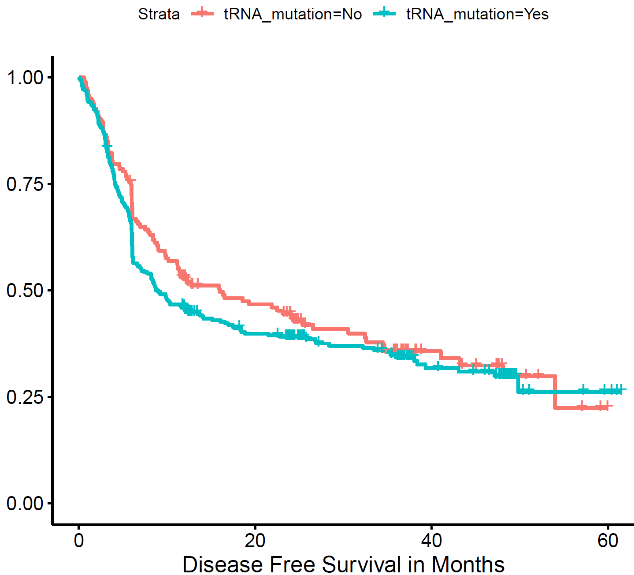

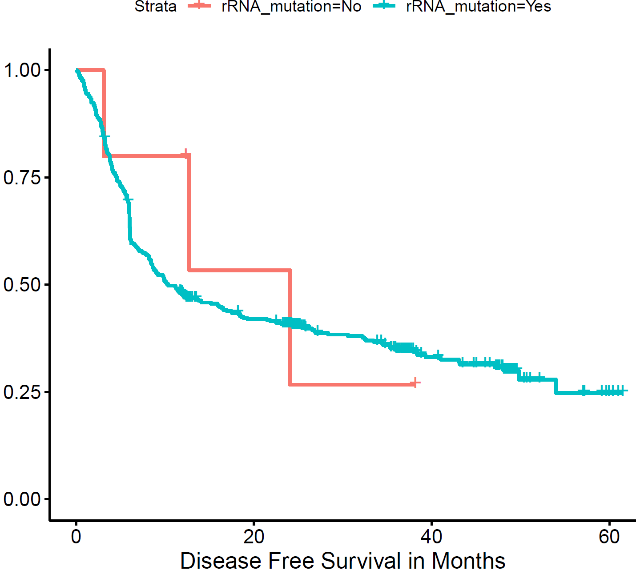


Survival probability

*CO3*

Log-Rank *P* = 0.11

*tRNA*

Log-Rank *P* = 0.28

*rRNA*

Log-Rank *P* = 0.74

**Supplementary Figure 4. Survival Curves for relapse based on mtDNA gene variants.**

Survival probability


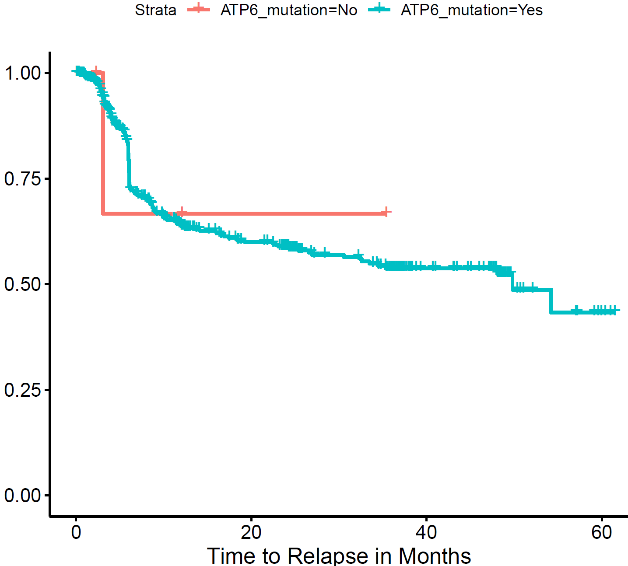

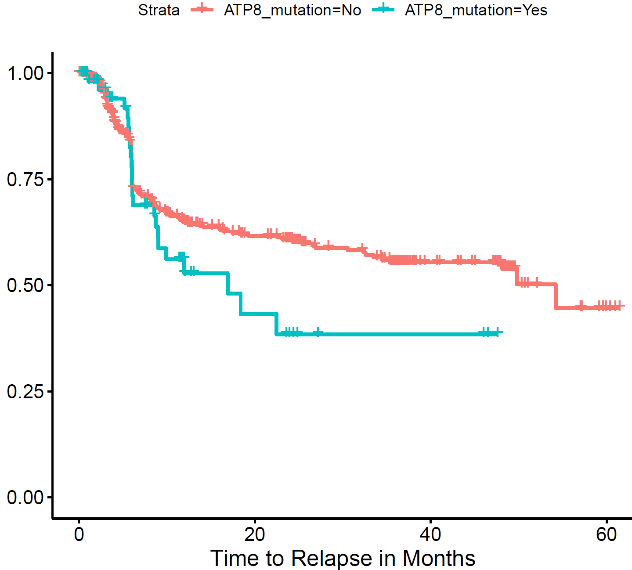

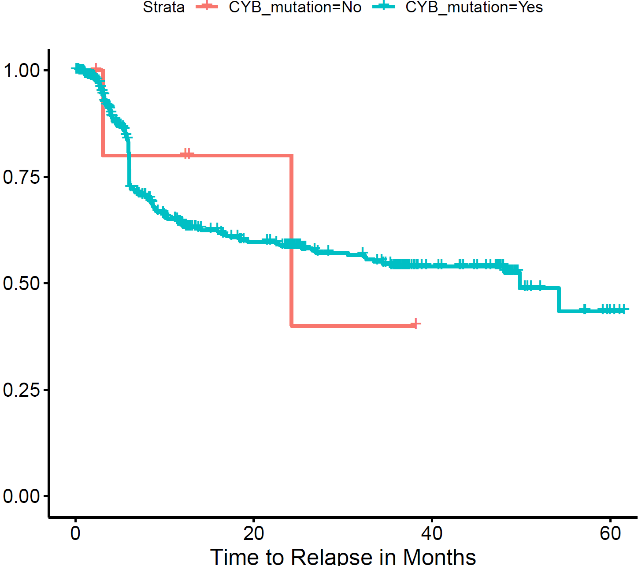


*ATP6*

Log-Rank *P* = 0.89

*ATP8*

Log-Rank *P* = 0.12

*CYB*

Log-Rank *P* = 0.93


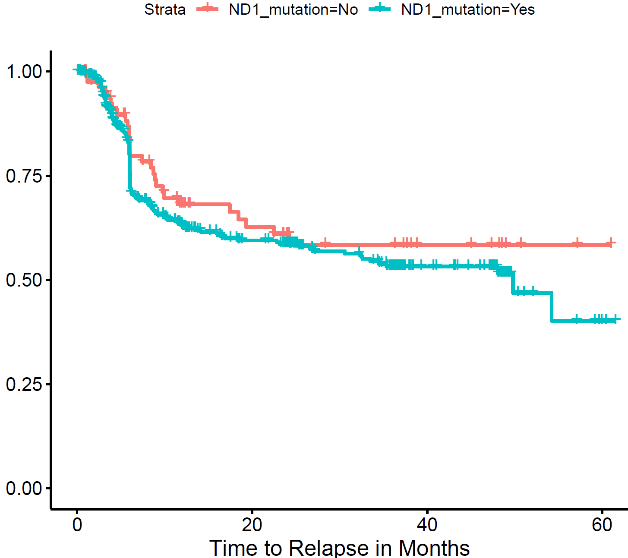

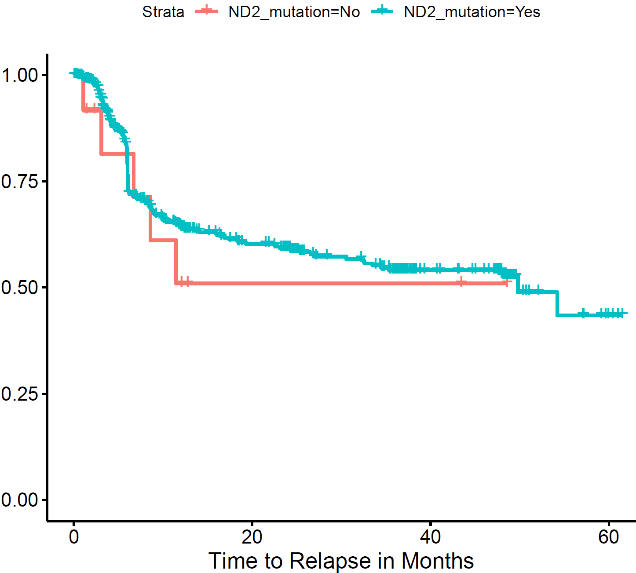

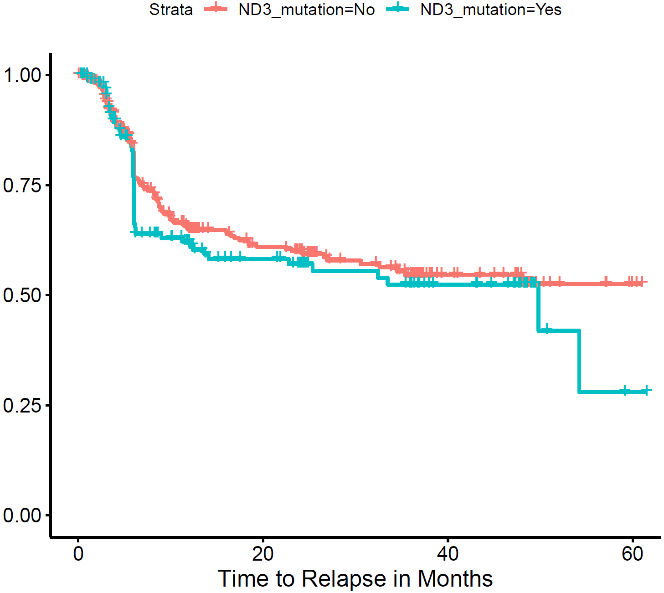


Survival probability

*ND1*

Log-Rank *P* = 0.38

*ND2*

Log-Rank *P* = 0.55

*ND3*

Log-Rank *P* = 0.34

Survival probability


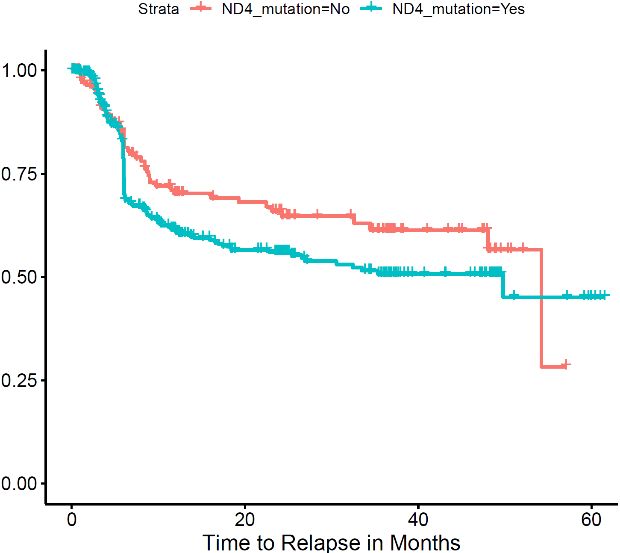

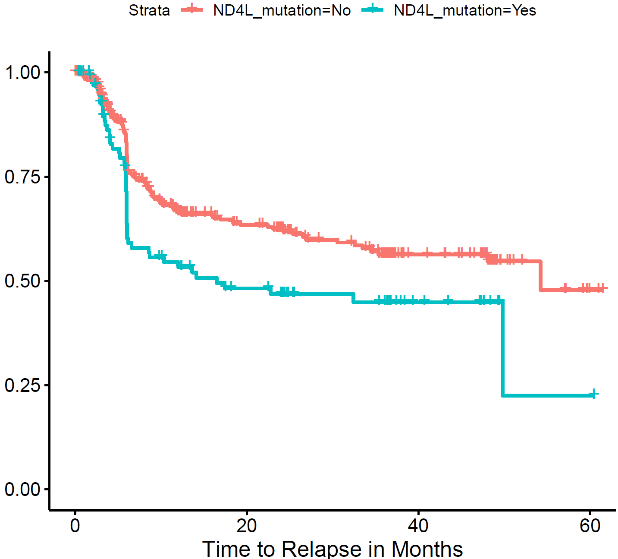

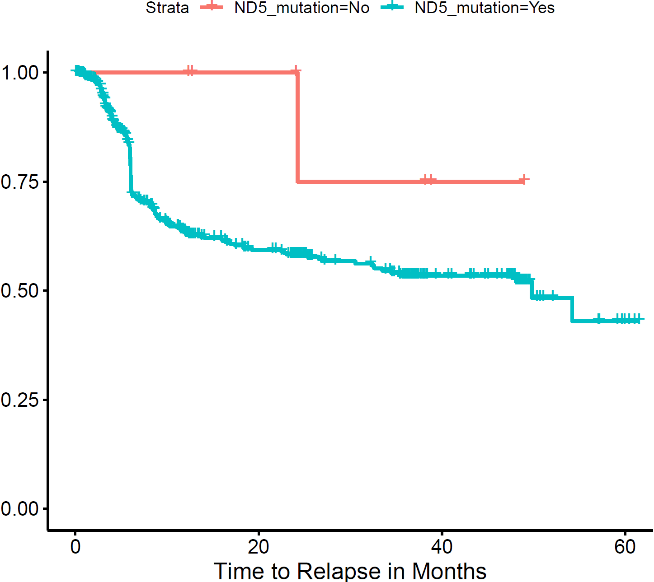


*ND5*

Log-Rank *P* = 0.14

*ND4*

Log-Rank *P* = 0.09

*ND4L*

Log-Rank *P* = 0.008

Survival probability


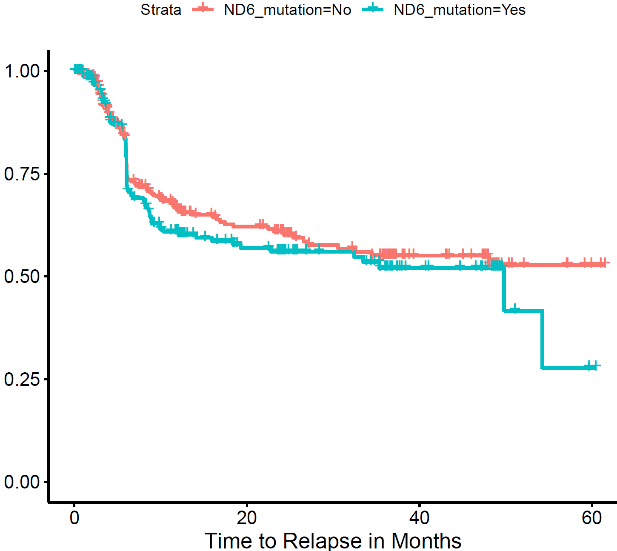

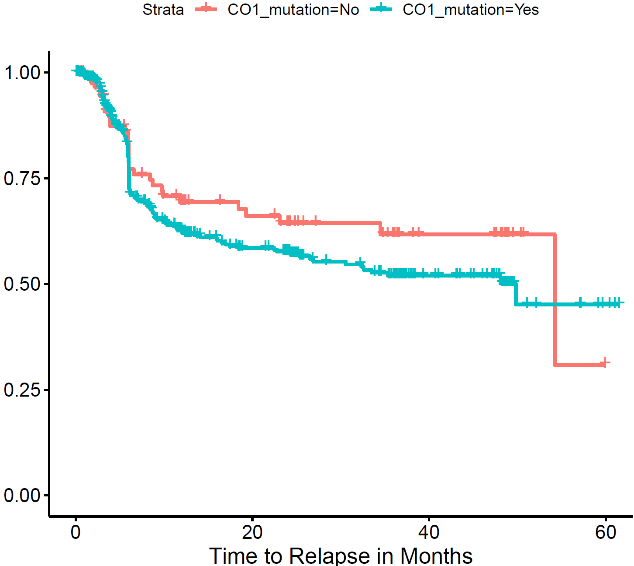

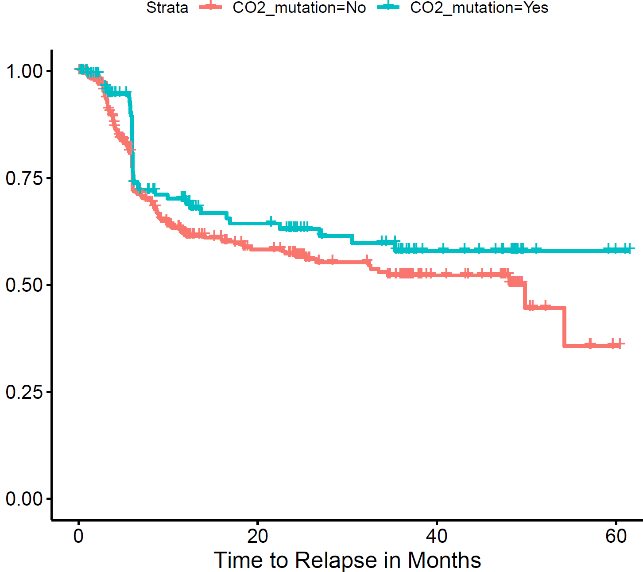


*ND6*

Log-Rank *P* = 0.35

*CO1*

Log-Rank *P* = 0.23

*CO2*

Log-Rank *P* = 0.13

Survival probability


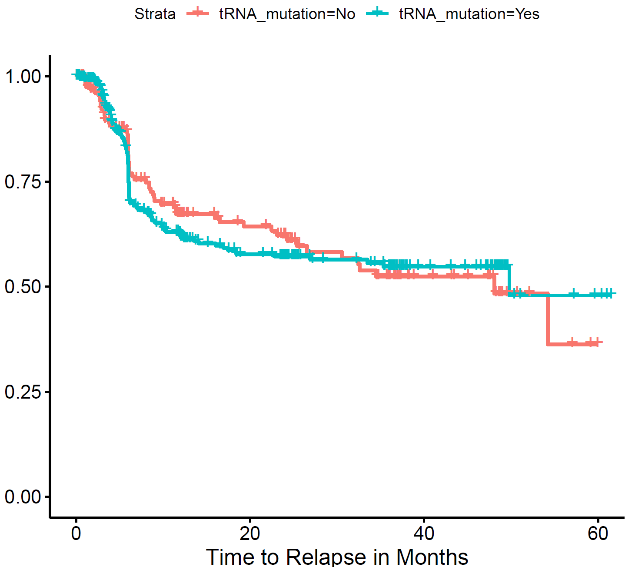

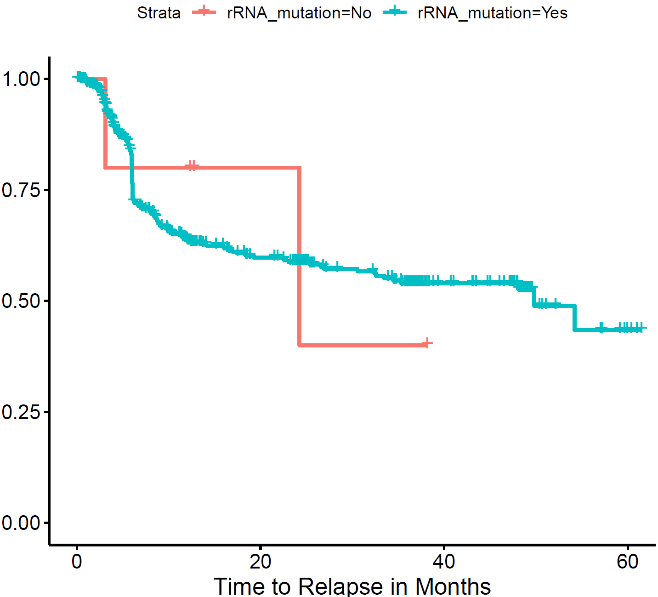

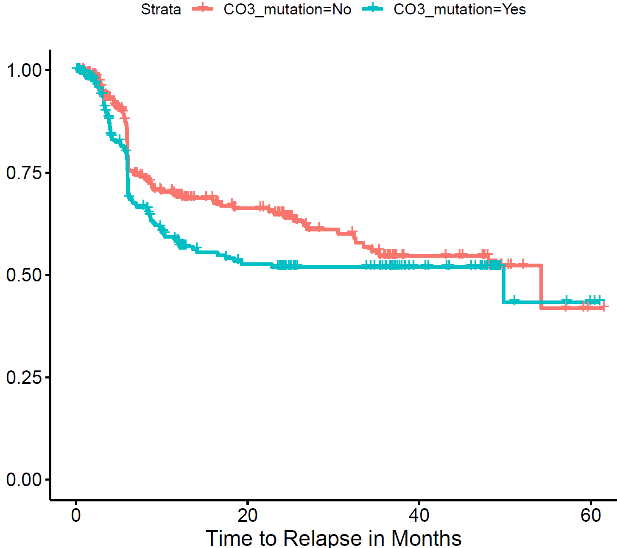


*rRNA*

Log-Rank *P* = 0.94

*tRNA*

Log-Rank *P* = 0.76

*CO3*

Log-Rank *P* = 0.08

**Supplementary Figure 5. Survival Curves for TRM based on mtDNA gene variants.**


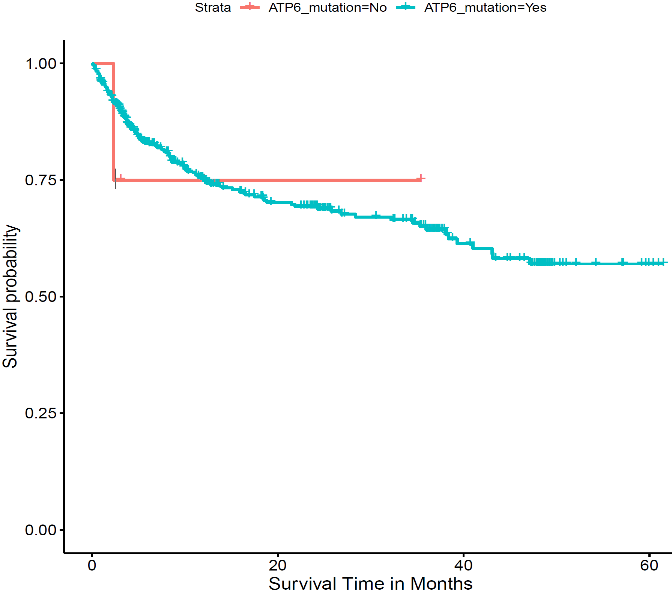


*ATP6*

Log-Rank *P* = 0.93


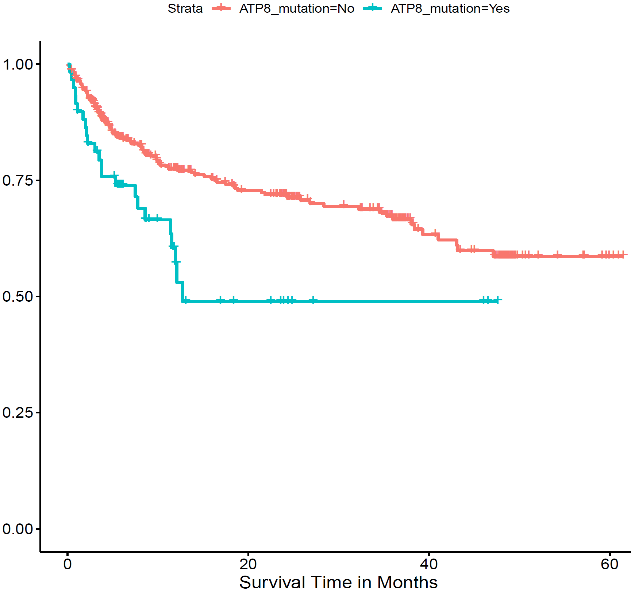


*ATP8*

Log-Rank *P* = 0.003


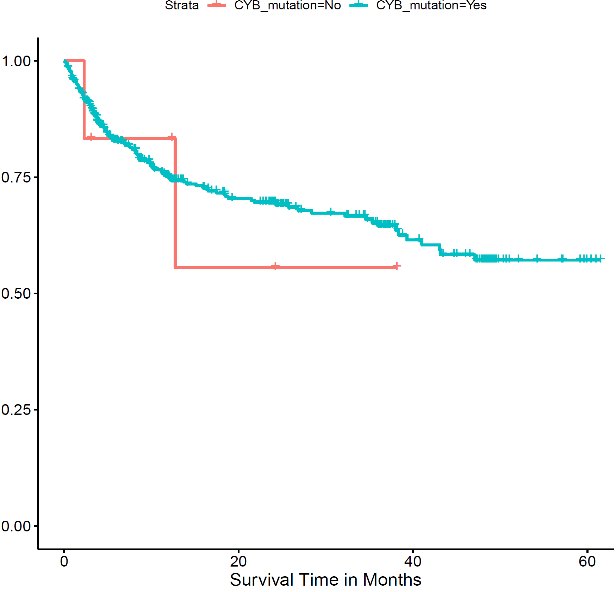


*CYB*

Log-Rank *P* = 0.76

Survival probability


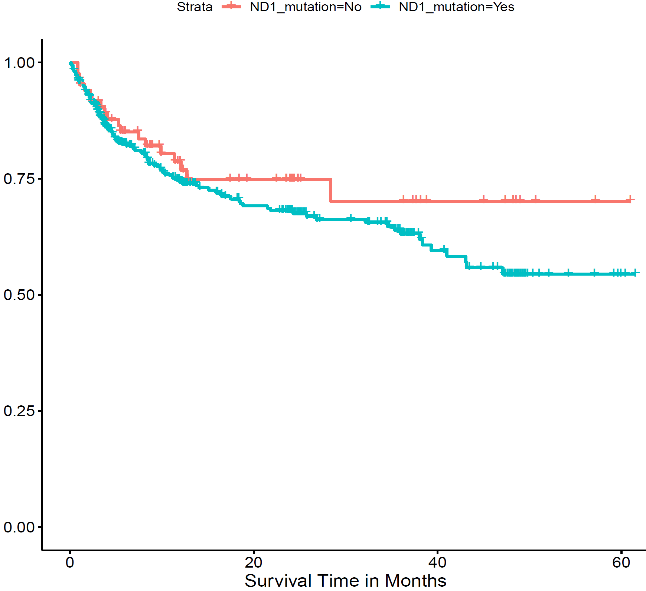


Survival probability

*ND1*

Log-Rank *P* = 0.23


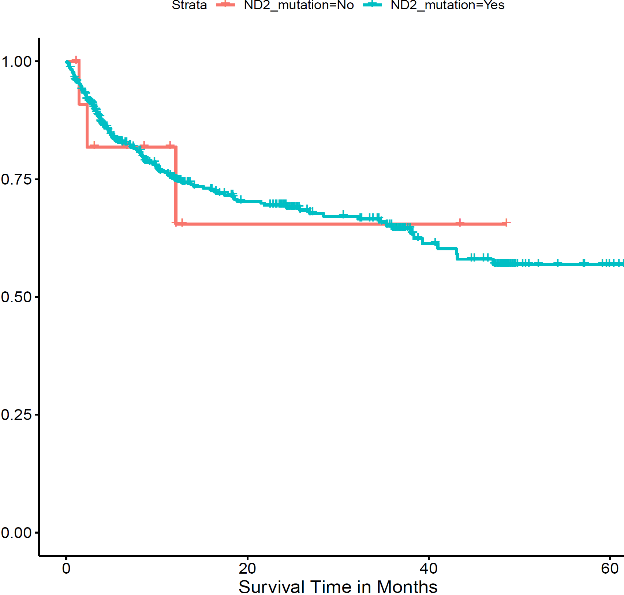


*ND2*

Log-Rank *P* = 0.99


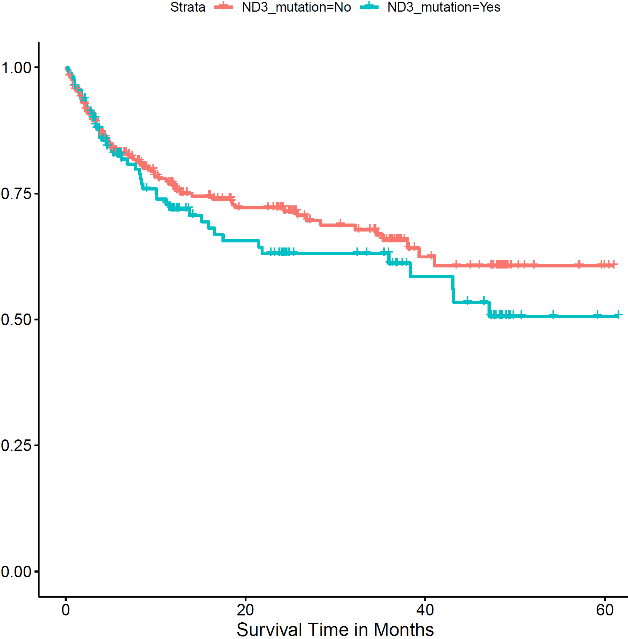


*ND3*

Log-Rank *P* = 0.28


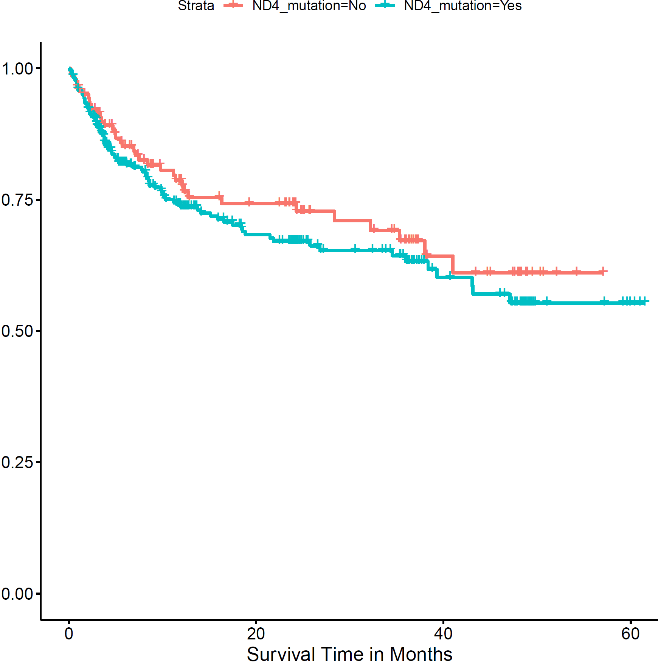


Survival probability

*ND4*

Log-Rank *P* = 0.31


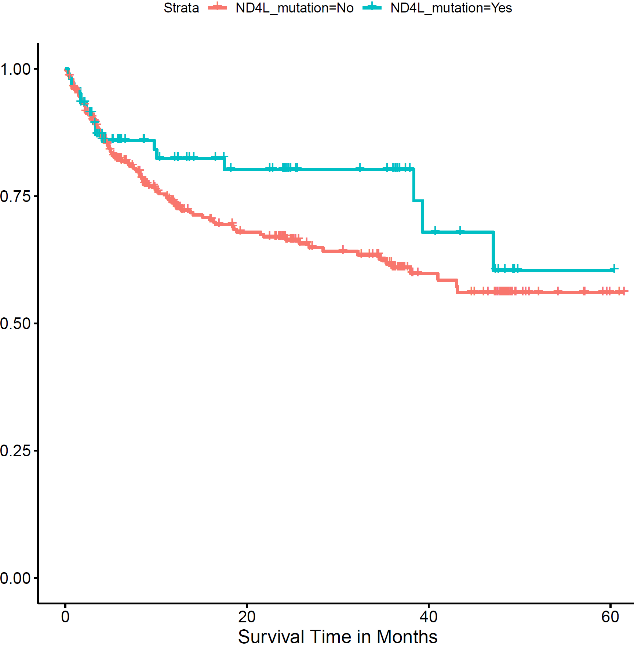


*ND4L*

Log-Rank *P* = 0.11


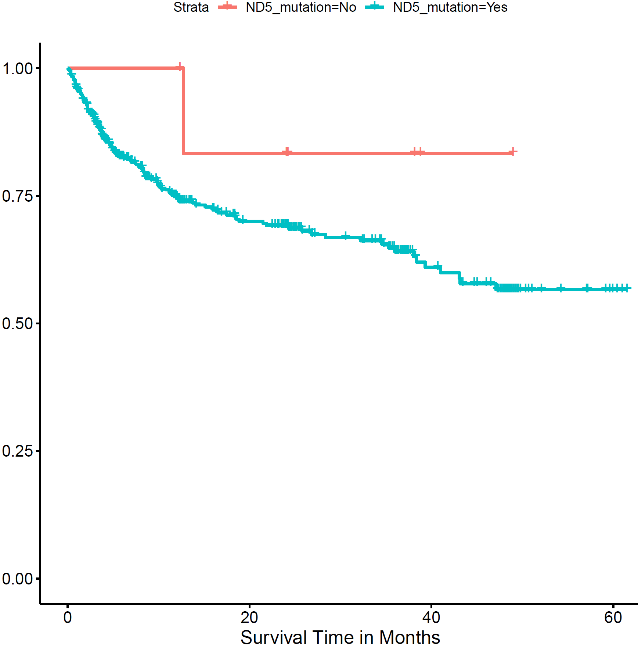


*ND5*

Log-Rank *P* = 0.28


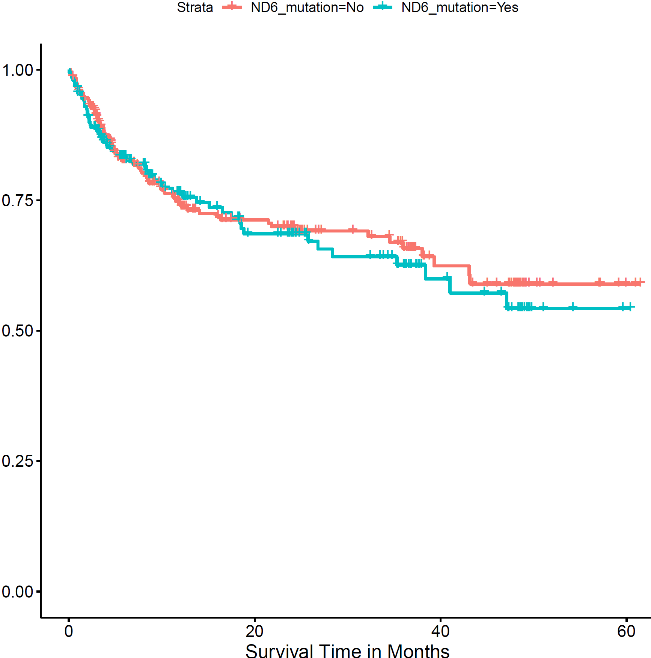


Survival probability

*ND6*

Log-Rank *P* = 0.73


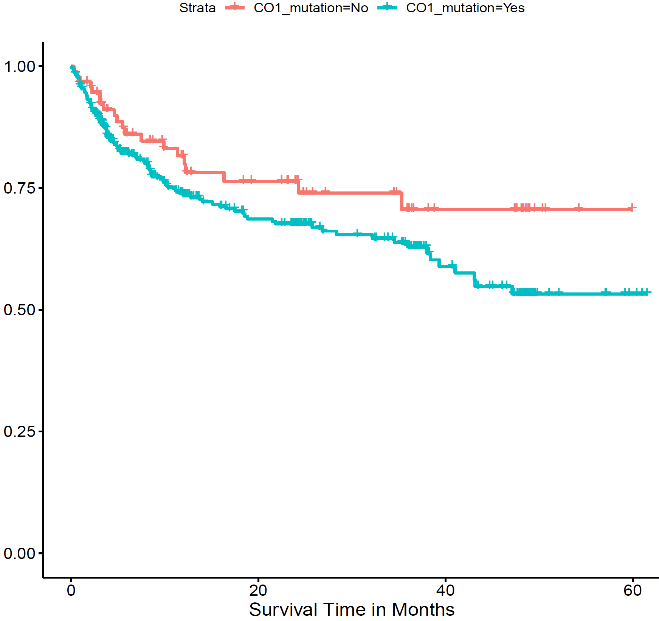


*CO1*

Log-Rank *P* = 0.09


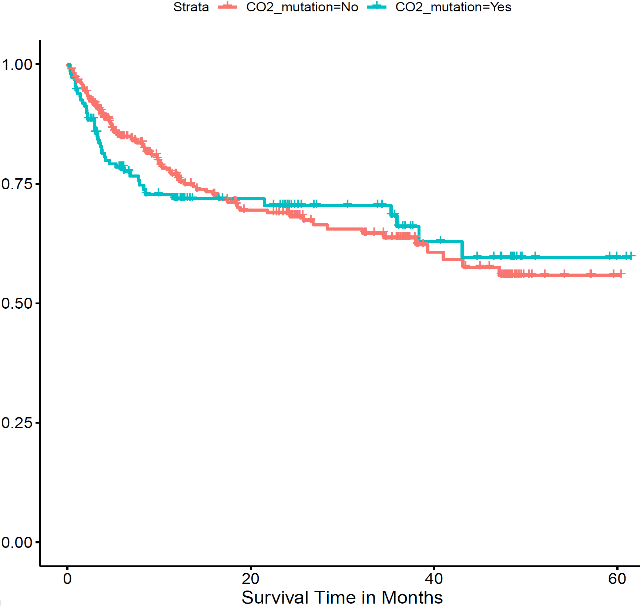


*CO2*

Log-Rank *P* = 0.64


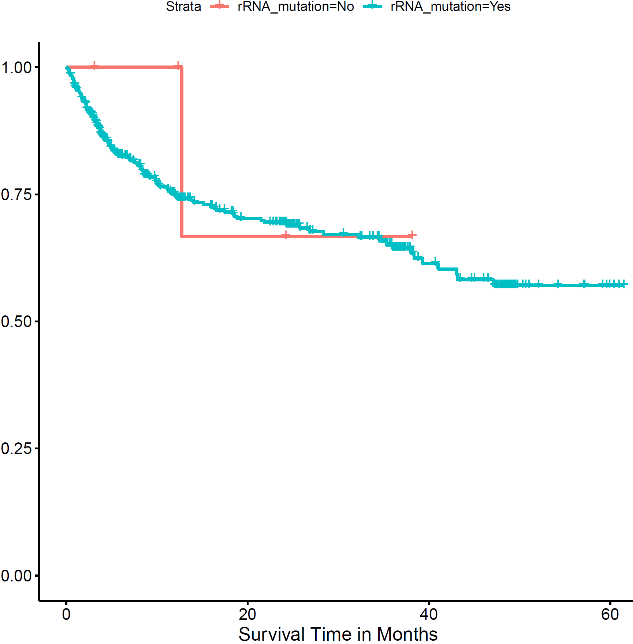

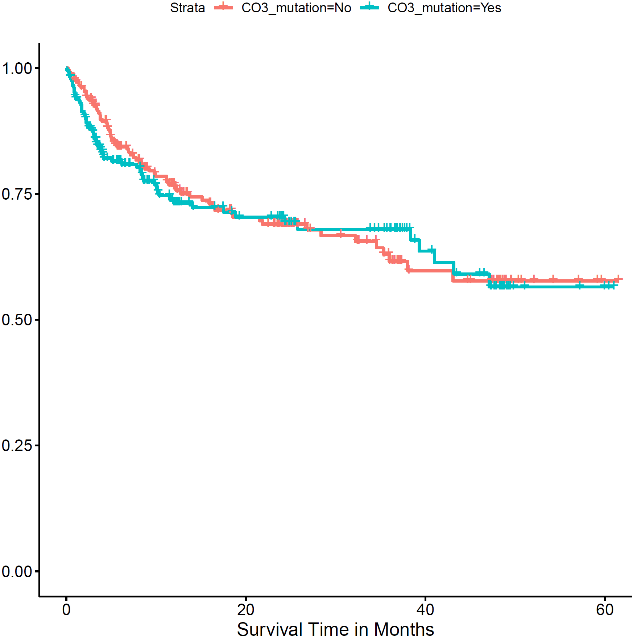


Survival probability

*CO3*

Log-Rank *P* = 0.68


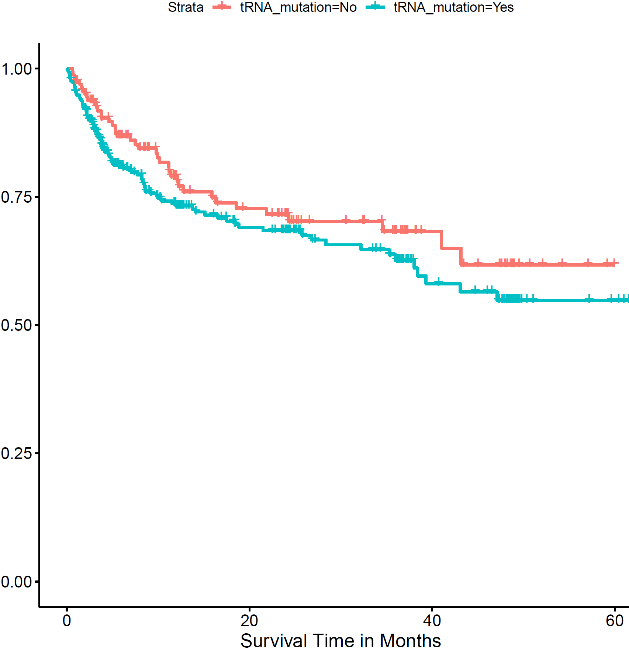


*tRNA*

Log-Rank *P* = 0.19

*rRNA*

Log-Rank *P* = 0.67

**Supplementary Figure 6. Important variables included in the final model.**


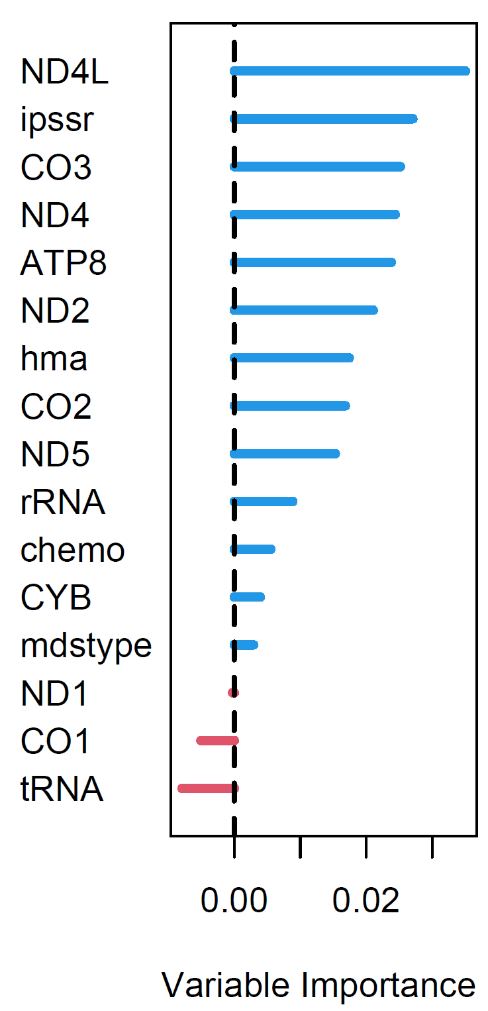

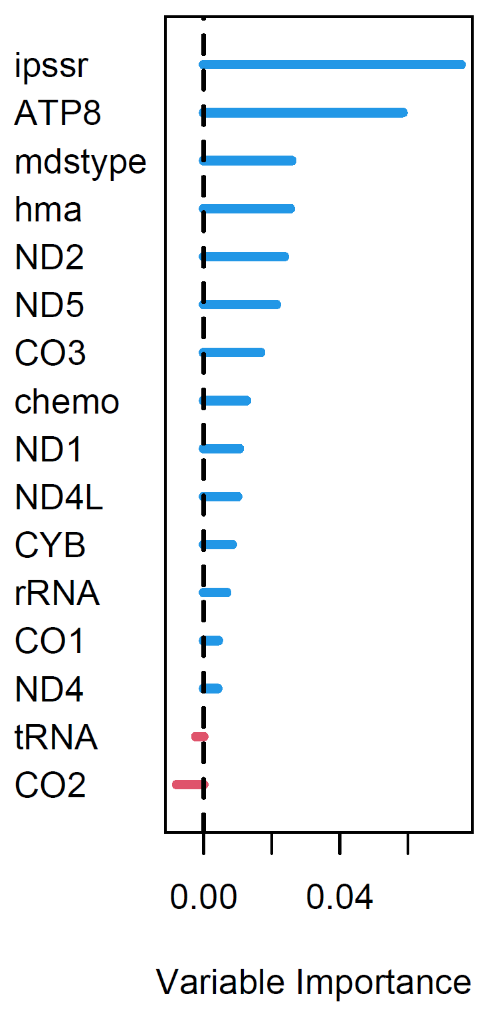


**Relapse**


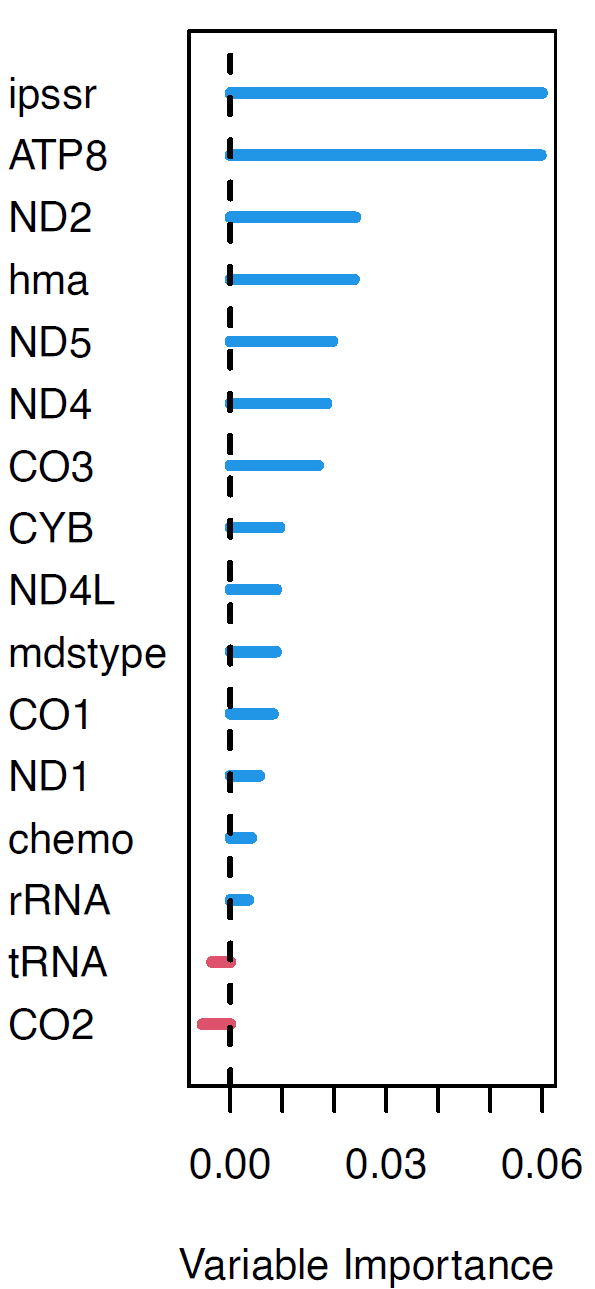

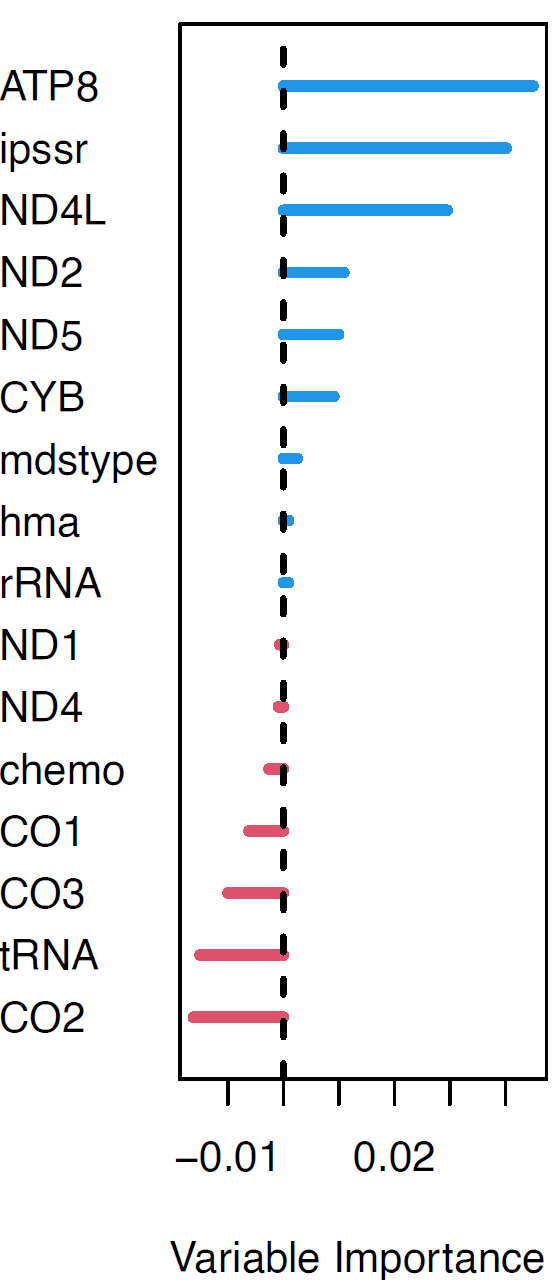


**OS**

**TRM**

**RFS**

**Supplementary Figure 7. Net reclassification improvement of mtDNA variants.** Categorization of patients according to the models based on the IPSS-R with and without adding mtDNA mutations. Y-axes represent the 1-, 2- and 5-year survival/relapse risk stratification based on IPSS-R score. Colored bars represent the model of “IPSS-R + mtDNA” risk stratification on the x-axis, within the stratification based only on the IPSS-R represented in rows. Numbers in each bar represent the number of patients reclassified by the model based on IPSS-R adding mtDNA mutations.

1. **OS**

**
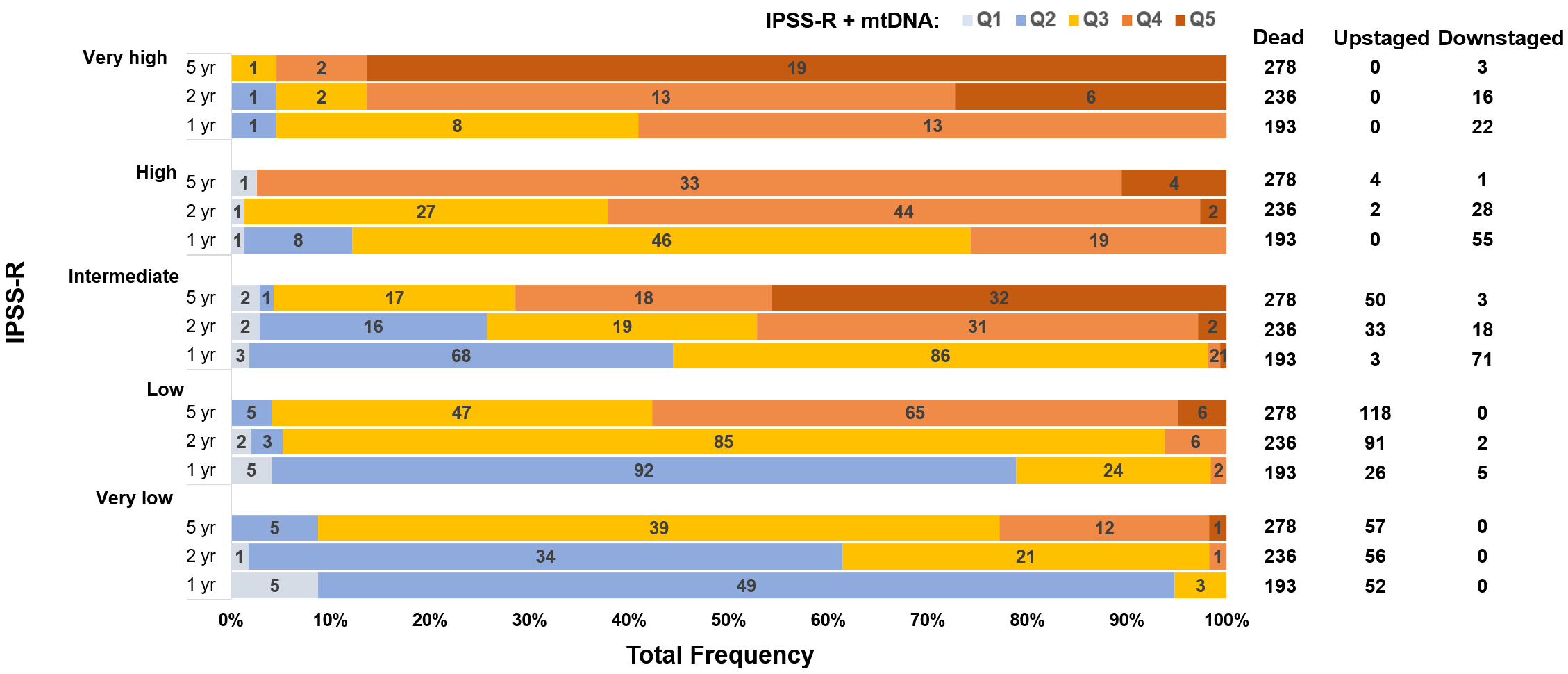
**

**
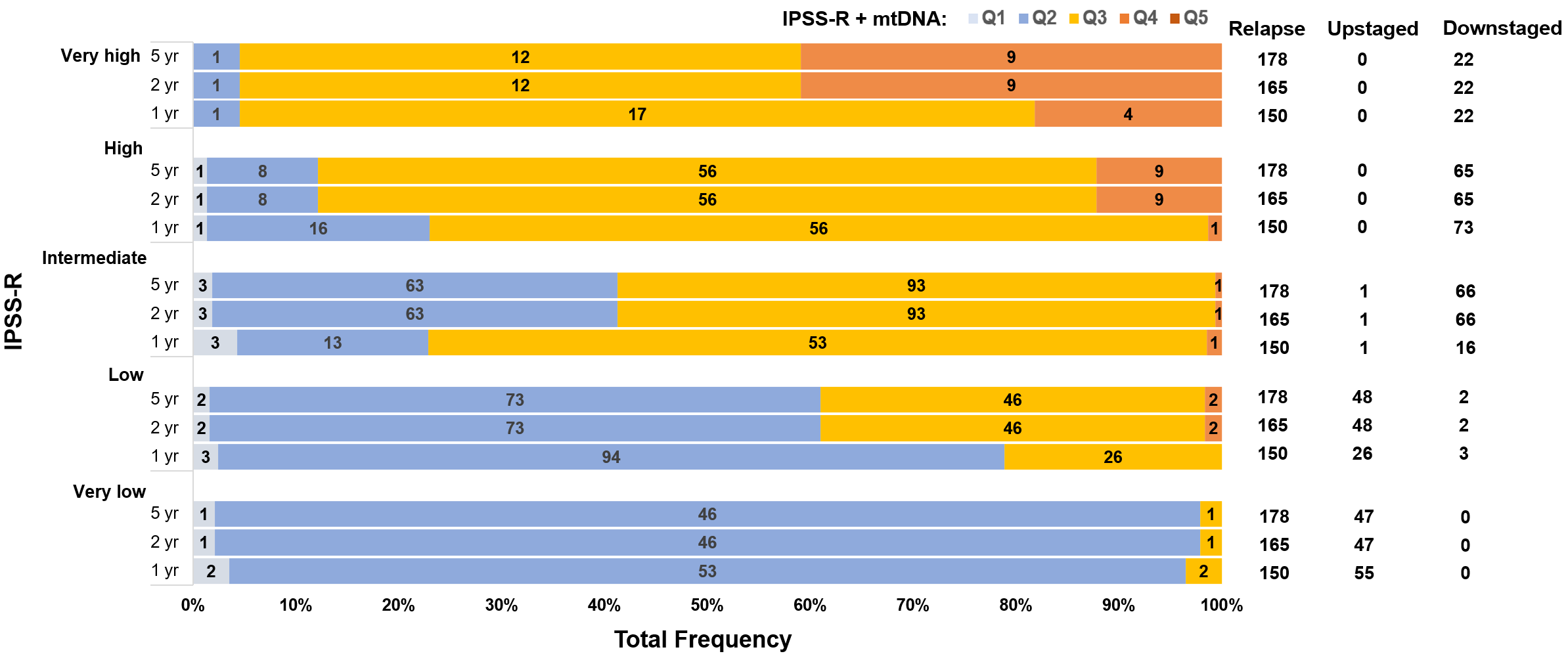
**

**B. Relapse**

**Supplementary Figure 8. Survival Curves of *TP53* mutations.**


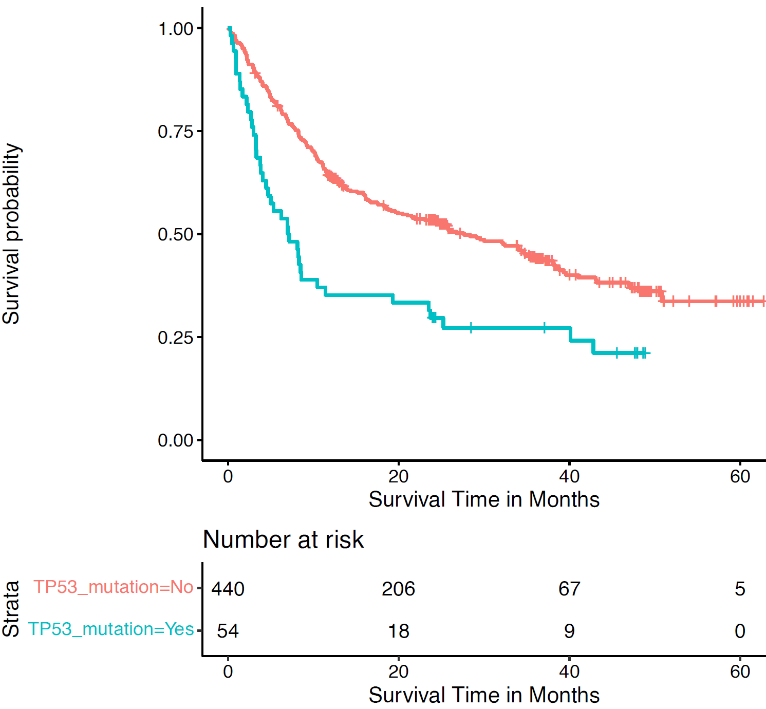

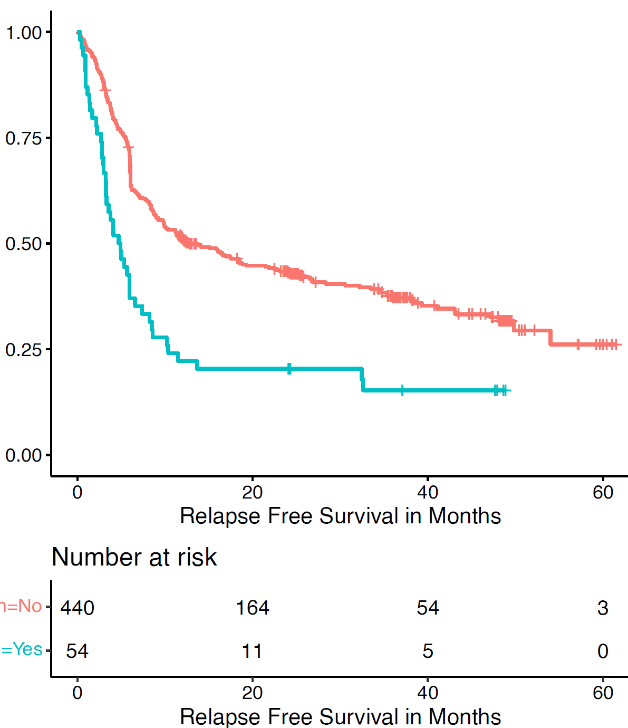

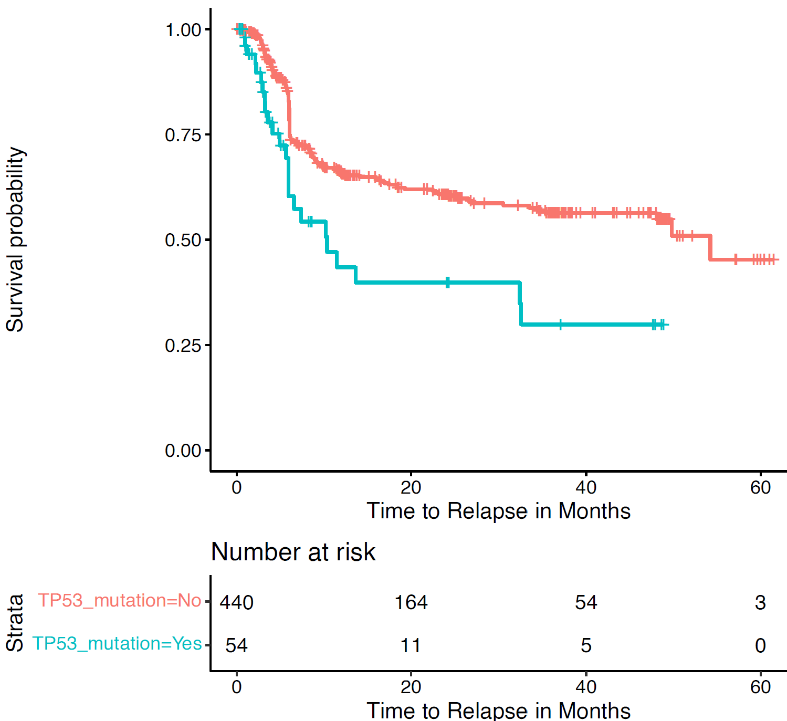

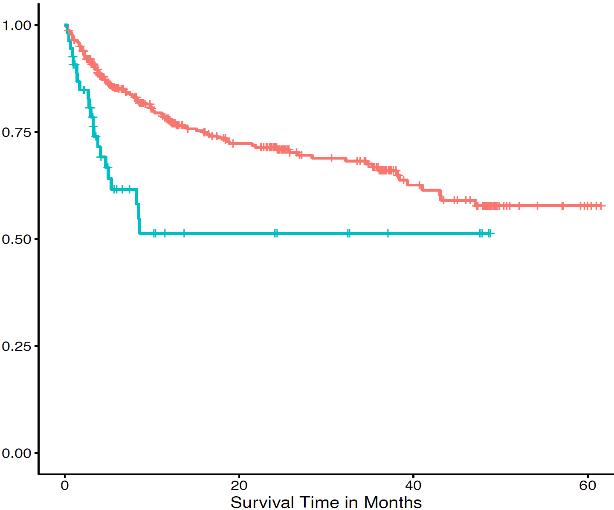

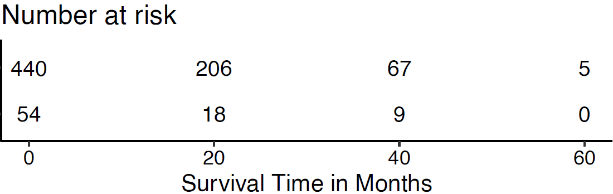

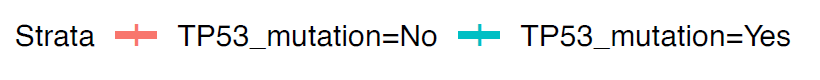


**OS**

**RFS**

**Relapse**

**TRM**

Log-Rank *P* = 6E-05

Log-Rank *P* = 2E-06

Log-Rank *P* = 6E-04

Log-Rank *P* = 0.001

**Supplementary Figure 9. Sequencing coverage of mitochondrial genome in MDS.** Top panel: mean read depth from 494 MDS patients (red line) with standard deviation of read depth (grey area). Bottom panel: A histogram representing the number of samples and their mean coverage across the mitochondrial genome.


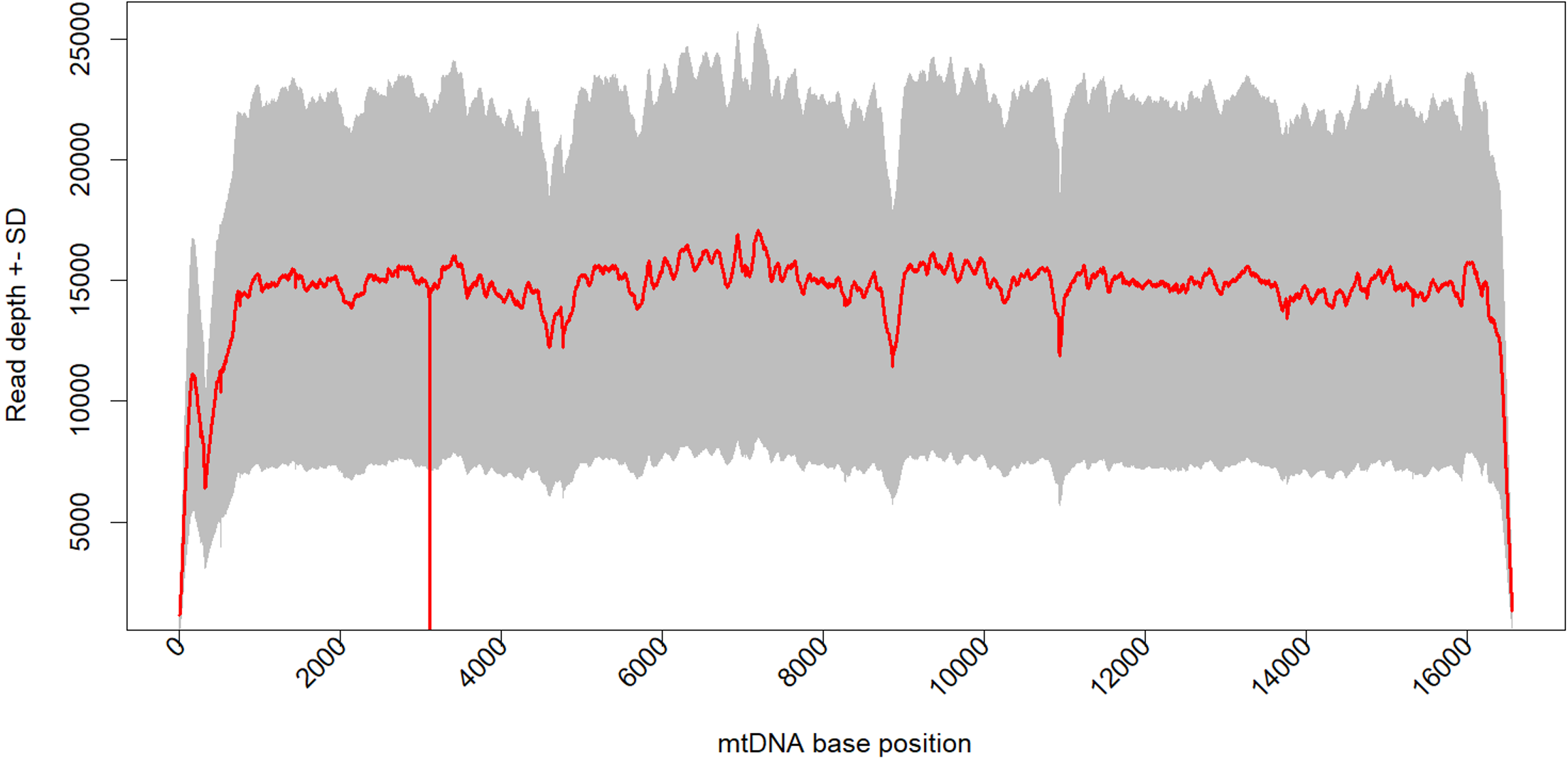


Read Depth + - SD

mtDNA Base Position


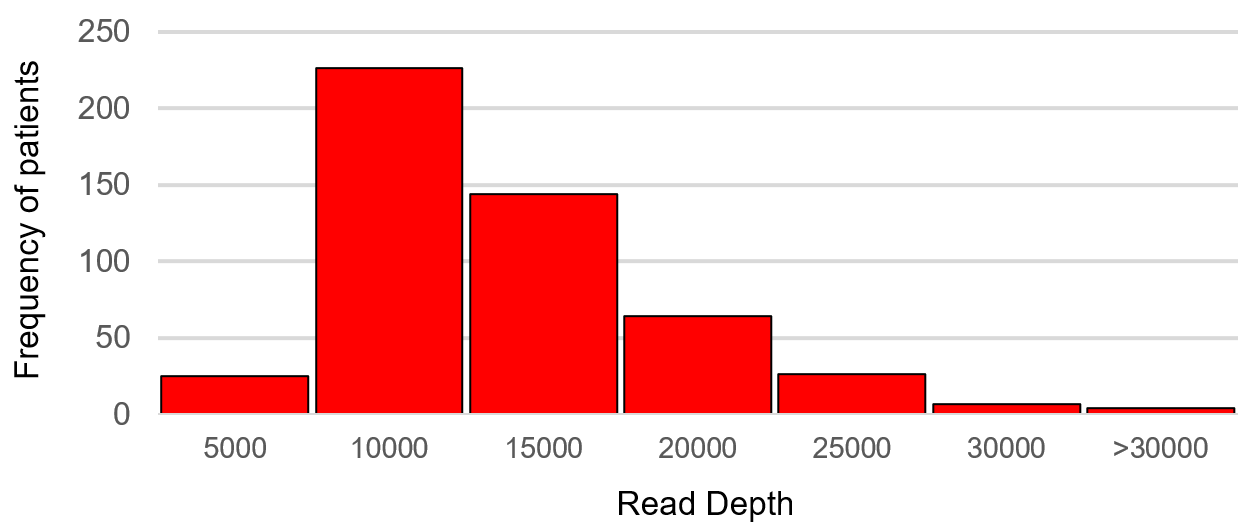

Supplement: Supplementary file 1 — Additional file 1. Supplementary Figures. [file 13045_2023_1418_MOESM1_ESM.docx]
