# Supplementary material for: Prognostic landscape of mitochondrial genome in myelodysplastic syndrome after stem-cell transplantation
Source: J Hematol Oncol. 2023 Mar 10;16:21. doi: 10.1186/s13045-023-01418-4 (PMC9999628; doi:10.1186/s13045-023-01418-4)
Supplement: Supplementary file 2 — Additional file 2. Supplementary Tables. [file 13045_2023_1418_MOESM2_ESM.docx]

**Supplementary Table 1. Characteristics of patients.**

| **Variables** | | **No. (%)** | **Variables** | | **No. (%)** |
| --- | --- | --- | --- | --- | --- |
| **Age, median (min-max)** | | 66.0 (22.6-78.6) | **MDS Cytogenetic score** | |  |
| **Sex** |  |  |  | Very good | 4 (1) |
|  | Male | 315 (64) |  | Good | 186 (38) |
| **Pre-transplant therapies** | |  |  | Intermediate | 109 (22) |
|  | HMA alone | 340 (69) |  | Poor | 67 (14) |
|  | Chemo alone | 15 (3) |  | Very poor | 126 (26) |
|  | Both | 34 (7) |  | Missing | 2 (0) |
|  | Neither | 93 (19) | **Cytogenetic abnormalities (yes/no)** | | |
|  | Missing | 12 (2) |  | Monosomy 5 | 16/229 |
| **Doner type** | |  |  | Monosomy 7 | 48/197 |
|  | HLA-identical sibling | 65 (13) |  | Monosomy 13 | 8/231 |
|  | Other related | 32 (6) |  | Monosomy 20 | 9/236 |
|  | Well-matched unrelated (8/8) | 353 (71) |  | Monosomy y | 9/236 |
|  | Partially matched unrelated (7/8) | 39 (8) |  | Trisomy 8 | 54/191 |
|  | Mis-matched unrelated (<=6/8) or other | 5 (1) |  | Trisomy 19 | 6/233 |
| **Regimen** | |  |  | Translocation *t*(1;3) | 3/236 |
|  | Myeloablative | 127 (26) |  | Translocation *t* (2;11) | 1/238 |
|  | Reduced intensity | 308 (62) |  | Translocation *t* (3;3) | 2/243 |
|  | Non-myeloablative | 44 (9) |  | Translocation *t* (3;21) | 3/236 |
|  | Missing | 15 (3) |  | Translocation *t* (6;9) | 0/245 |
| **Graft type** | |  |  | Translocation *t* (11;16) | 0/239 |
|  | Bone Marrow | 59 (12) |  | Deletion del(3q) | 7/232 |
|  | Peripheral blood | 435 (88) |  | Deletion del(5q) | 64/181 |
| **MDS IPSS-R score pre-transplant** | | |  | Deletion del(7q) | 38/207 |
|  | Very low | 57 (12) |  | Deletion del(9q) | 4/241 |
|  | Low | 123 (25) |  | Deletion del(11q) | 5/240 |
|  | Intermediate | 160 (32) |  | Deletion del(12q) | 11/228 |
|  | High | 74 (15) |  | Deletion del(13q) | 8/231 |
|  | Very high | 22 (4) |  | Deletion del(20q) | 27/218 |
|  | Missing | 58 (12) |  | Inversion inv(3) | 3/242 |
| **MDS HCT-CI score** | |  | **Karnofsky performance status** | | |
|  | Low risk | 61 (12) |  | 10-80 | 251 (51) |
|  | Intermediate risk | 110 (22) |  | 90-100 | 243 (45) |
|  | High risk | 315 (64) | **Time from diagnosis to HCT (mo.) median (range)** | | |
|  | Missing | 8 (2) |  |  | 18 (2-263) |

**Supplementary Table 2. Associations of putative pathogenic variants**

| **Variant** | **Gene** | **N** | **Type** | **Amio Acid Change** | **HF** | **OS** | | **RFS** | | **Relapse** | | **TRM** | |
| --- | --- | --- | --- | --- | --- | --- | --- | --- | --- | --- | --- | --- | --- |
|  |  |  |  |  |  | **HR** | ***P*** | **HR** | ***P*** | **HR** | ***P*** | **HR** | ***P*** |
| m.8837T>C | *ATP6* | 1 | Missense | M104T | 0.092 | 95.76 | 2.76E-05* | 62.48 | 1.22E-04* | 1.43E-04 | 9.97E-01 | 84.62 | 8.53E-05* |
| m.8939T>C | *ATP6* | 2 | Missense | I138T | 0.505 | 5.33 | 2.12E-02 | 6.11 | 1.31E-02 | 19.22 | 9.87E-05* | 1.69E-06 | 9.93E-01 |
| m.6036G>A | *CO1* | 1 | Missense | G45S | 0.682 | 19.73 | 4.53E-03 | 28.79 | 1.38E-03 | 196.40 | 6.21E-06* | 7.09E-06 | 9.95E-01 |
| m.6168G>A | *CO1* | 2 | Missense | A89T | 0.019 | 4.19 | 5.86E-02 | 9.43 | 3.11E-03 | 48.66 | 2.05E-06* | 1.70E-06 | 9.93E-01 |
| m.6711T>C | *CO1* | 1 | Missense | Y270H | 0.027 | 12.56 | 1.66E-02 | 108.42 | 2.44E-05* | NA | NA | 1.47E-05 | 9.96E-01 |
| m.7191T>C | *CO1* | 1 | Missense | F430L | 0.179 | 12.92 | 1.75E-02 | 14.70 | 1.21E-02 | 180.88 | 6.17E-05* | 2.61E-06 | 9.95E-01 |
| m.9307G>A | *CO3* | 1 | Stop-gain | Stop-gain | 0.032 | 7.36 | 5.17E-02 | 14.43 | 9.76E-03 | 75.35 | 1.12E-04* | 4.36E-06 | 9.95E-01 |
| m.15173G>A | *CYB* | 1 | Missense | A143T | 0.010 | 142.27 | 1.09E-04* | 93.32 | 2.74E-04 | 1.47E-04 | 9.97E-01 | 189.88 | 1.36E-03 |
| m.15756G>A | *CYB* | 1 | Stop-gain | Stop-gain | 0.042 | 142.27 | 1.09E-04* | 93.32 | 2.74E-04 | 1.47E-04 | 9.97E-01 | 189.88 | 1.36E-03 |
| m.4211T>C | *ND1* | 1 | Missense | M302T | 0.010 | 147.96 | 1.49E-05* | 176.49 | 6.91E-06* | NA | NA | 175.00 | 1.32E-05* |
| m.10554T>C | *ND4L* | 1 | Missense | S29P | 0.022 | 14.81 | 9.08E-03 | 13.64 | 1.11E-02 | 72.30 | 1.07E-04* | 6.14E-06 | 9.94E-01 |
| m.12704T>C | *ND5* | 1 | Missense | I123T | 0.054 | 12.92 | 1.75E-02 | 14.70 | 1.21E-02 | 180.88 | 6.17E-05* | 2.61E-06 | 9.95E-01 |
| m.12769G>A | *ND5* | 1 | Missense | E145K | 0.059 | 12.56 | 1.66E-02 | 108.42 | 2.44E-05* | NA | NA | 1.47E-05 | 9.96E-01 |

Models are adjusted for age at transplantation, IPSS-R score, MDS type, pre-HCT treatments and first 10 principal components.

HF: heteroplasmic fraction.

*Significant *P* values after Bonferroni correction. Due to the small number of patients carrying these mutations, the significance may not be robust.

**Supplementary Table 3. Associations between common variants and overall survival .**

| **Variant** | **Gene** | **N** | **dbSNP** | **Type** | **Amio Acid Change** | **Multivariable analysis** | | **Conditional analysis** | |
| --- | --- | --- | --- | --- | --- | --- | --- | --- | --- |
|  |  |  |  |  |  | **HR (95% CI)** | ***P*** | **HR (95% CI)** | ***P*** |
| m.204T>C | *D-Loop* | 60 | rs3135032 | Non-coding |  | 0.52 (0.31-0.88) | 1.54E-02 | 0.57 (0.34-0.96) | 3.55E-02 |
| m.319T>C | *D-Loop* | 9 | rs879055956 | Non-coding |  | 0.16 (0.03-0.86) | 3.27E-02 | NA |  |
| m.477T>C | *D-Loop* | 17 | rs41442247 | Non-coding |  | 0.40 (0.17-0.91) | 2.96E-02 | 0.42 (0.18-0.99) | 4.78E-02 |
| m.16051A>G | *D-Loop* | 8 | rs117565943 | Non-coding |  | 2.78 (1.15-6.73) | 2.30E-02 | 2.57 (0.92-7.13) | 7.08E-02 |
| m.16186C>T | *D-Loop* | 17 | rs879166752 | Non-coding |  | 2.51 (1.29-4.87) | 6.72E-03 | 3.51 (0.95-12.88) | 5.89E-02 |
| m.16163A>G | *D-Loop* | 15 | rs41479950 | Non-coding |  | 2.17 (1.02-4.64) | 4.54E-02 | 1.24 (0.15-10.05) | 8.43E-01 |
| m.16293A>G | *D-Loop* | 11 |  | Non-coding |  | 2.89 (1.5-5.58) | 1.50E-03 | 1.91 (0.89-4.09) | 9.66E-02 |
| m.16294C>T | *D-Loop* | 60 | rs140662392 | Non-coding |  | 2.69 (1.00-7.25) | 5.00E-02 | 1.10 (0.37-3.26) | 8.62E-01 |
| m.16231T>C | *D-Loop* | 9 | rs386829286 | Non-coding |  | 0.16 (0.03-0.86) | 3.27E-02 | NA |  |
| m.16255G>A | *D-Loop* | 5 | rs1603225769 | Non-coding |  | 3.81 (1.29-11.28) | 1.57E-02 | 4.08 (1.22-13.57) | 2.20E-02 |
| m.7789G>A | *CO2* | 9 | rs386829014 | Synonymous |  | 0.16 (0.03-0.86) | 3.27E-02 | NA |  |
| m.9656T>C | *CO3* | 6 | rs1556423706 | Synonymous | S150S | 5.79 (2.29-14.62) | 2.01E-04 | 6.27 (2.3-17.04) | 3.25E-04* |
| m.10463T>C | *TR* | 57 | rs28358279 | tRNA |  | 0.11 (0.02-0.52) | 5.13E-03 | 0.13 (0.03-0.59) | 8.04E-03 |
| m.961T>G | *RNR1* | 5 | rs3888511 | rRNA |  | 3.16 (1.27-7.89) | 1.37E-02 | 2.63 (0.96-7.24) | 6.09E-02 |
| m.8448T>C | *ATP8* | 5 | rs879056797 | Missense | M28T | 3.16 (1.27-7.89) | 1.37E-02 | NA |  |
| m.8512A>G | *ATP8* | 5 | rs1556423477 | Synonymous | K49K | 2.93 (1.04-8.24) | 4.14E-02 | 3.08 (1.09-8.76) | 3.45E-02 |
| m.5495T>C | *ND2* | 9 | rs3020602 | Synonymous | F342F | 2.98 (1.26-7.04) | 1.30E-02 | 3.75 (1.53-9.22) | 3.94E-03 |
| m.10499A>G | *ND4L* | 11 | rs1057520074 | Synonymous | L10L | 0.19 (0.04-0.84) | 2.84E-02 | 0.61 (0.07-5.15) | 6.47E-01 |
| m.10550A>G | *ND4L* | 47 | rs28358280 | Synonymous | M27M | 0.16 (0.03-0.75) | 1.99E-02 | 0.61 (0.08-4.52) | 6.32E-01 |
| m.11031GA>G* | *ND4* | 24 | rs1556423884 | Frame_Shift |  | 0.28 (0.12-0.69) | 5.46E-03 | 0.33 (0.13-0.82) | 1.66E-02 |
| m.11812A>G | *ND4* | 39 | rs3088053 | Synonymous | L351L | 0.38 (0.17-0.86) | 1.93E-02 | NA |  |
| m.12633C>A | *ND5* | 16 | rs3926883 | Synonymous | S99S | 2.18 (1.04-4.56) | 3.85E-02 | 0.80 (0.02-33.01) | 9.06E-01 |
| m.13722A>G | *ND5* | 10 | rs386829190 | Synonymous | L462L | 0.13 (0.03-0.67) | 1.44E-02 | NA |  |
| m.13934C>T | *ND5* | 16 | rs193302971 | Missense | T533M | 0.34 (0.14-0.82) | 1.58E-02 | 0.29 (0.12-0.7) | 5.68E-03 |
| m.14233A>G | *ND6* | 40 | rs3915611 | Synonymous | D147D | 0.37 (0.17-0.8) | 1.20E-02 | NA |  |
| m.14470T>A | *ND6* | 7 | rs3135030 | Synonymous | G68G | 2.37 (1.01-5.55) | 4.74E-02 | 2.49 (1.05-5.88) | 3.79E-02 |
| m.15784T>C* | *CYB* | 5 | rs527236194 | Synonymous | P346P | 3.14 (1.03-9.61) | 4.45E-02 | 2.49 (0.73-8.51) | 1.45E-01 |

D-Loop: control region. Models are adjusted for age at transplantation, IPSS-R score, MDS type, pre-HCT treatments and first 10 principal components.

*Significant *P* values after Bonferroni correction.

**Supplementary Table 4. Associations between common variants and relapse free survival.**

| **Variant** | **Gene** | **N** | **dbSNP** | **Type** | **Amio Acid Change** | **Multivariable analysis** | | **Conditional analysis** | |
| --- | --- | --- | --- | --- | --- | --- | --- | --- | --- |
|  |  |  |  |  |  | **HR (95% CI)** | ***P*** | **HR (95% CI)** | ***P*** |
| m.8448T>C | *ATP8* | 5 | rs879056797 | Missense | M28T | 2.50 (1.00-6.20) | 4.88E-02 | NA |  |
| m.9477G>A | *CO3* | 51 | rs2853825 | Missense | V91I | 0.28 (0.09-0.89) | 3.12E-02 | NA |  |
| m.9656T>C | *CO3* | 6 | rs1556423706 | Synonymous | S150S | 3.88 (1.55-9.72) | 3.86E-03 | 3.48 (0.39-31.22) | 2.66E-01 |
| m.16051A>G | *D-Loop* | 8 | rs117565943 | Non-coding | | 2.86 (1.27-6.42) | 1.09E-02 | 2.76 (1.08-7.04) | 3.39E-02 |
| m.16183A>C | *D-Loop* | 65 |  | Non-coding | | 0.42 (0.20-0.91) | 2.82E-02 | 0.38 (0.15-0.97) | 4.23E-02 |
| m.16222C>T | *D-Loop* | 8 | rs386829282 | Non-coding | | 2.32 (1.01-5.33) | 4.67E-02 | 1.11 (0.26-4.75) | 8.91E-01 |
| m.16255G>A | *D-Loop* | 5 | rs1603225769 | Non-coding |  | 2.90 (1.01-8.35) | 4.81E-02 | 1.80 (0.44-7.29) | 4.11E-01 |
| m.16293A>G | *D-Loop* | 11 |  | Non-coding | | 2.74 (1.42-5.29) | 2.55E-03 | 1.62 (0.74-3.52) | 2.27E-01 |
| m.16343A>G | *D-Loop* | 6 | rs374065731 | Non-coding | | 3.08 (1.22-7.75) | 1.68E-02 | NA |  |
| m.189A>G | *D-Loop* | 25 | rs371543232 | Non-coding | | 0.42 (0.18-0.95) | 3.64E-02 | 1.04 (0.46-2.34) | 9.19E-01 |
| m.204T>C | *D-Loop* | 60 | rs3135032 | Non-coding | | 0.55 (0.34-0.90) | 1.65E-02 | 0.65 (0.4-1.05) | 7.91E-02 |
| m.225G>A | *D-Loop* | 5 | rs372946833 | Non-coding | | 3.33 (1.25-8.85) | 1.58E-02 | 1.79 (0.47-6.85) | 3.97E-01 |
| m.242C>T | *D-Loop* | 5 | rs1556422403 | Non-coding | | 3.22 (1.19-8.72) | 2.11E-02 | 4.40 (0.78-24.86) | 9.37E-02 |
| m.477T>C | *D-Loop* | 17 | rs41442247 | Non-coding | | 0.39 (0.18-0.85) | 1.79E-02 | 0.43 (0.19-0.95) | 3.73E-02 |
| m.15454T>C | *CYB* | 6 | rs879015290 | Synonymous | L236L | 3.08 (1.22-7.75) | 1.68E-02 | NA |  |
| m.5495T>C | *ND2* | 9 | rs3020602 | Synonymous | F342F | 2.94 (1.24-6.93) | 1.39E-02 | 4.31 (1.74-10.67) | 1.62E-03* |
| m.11251A>G | *ND4* | 108 | rs869096886 | Synonymous | L164L | 0.21 (0.05-0.98) | 4.72E-02 | 0.55 (0.09-3.29) | 5.09E-01 |
| m.11485T>C | *ND4* | 8 | rs28529320 | Synonymous | G242G | 0.35 (0.14-0.90) | 2.87E-02 | 0.40 (0.15-1.08) | 6.97E-02 |
| m.10550A>G | *ND4L* | 47 | rs28358280 | Synonymous | M27M | 0.15 (0.03-0.63) | 9.69E-03 | 1.02 (0.15-6.73) | 9.83E-01 |
| m.13263A>G | *ND5* | 6 | rs28359175 | Synonymous | Q309Q | 3.47 (1.32-9.08) | 1.14E-02 | 2.60 (0.9-7.53) | 7.76E-02 |
| m.14139A>G | *ND5* | 6 | rs878918283 | Synonymous | L601L | 3.08 (1.22-7.75) | 1.68E-02 | 0.91 (0.09-9.2) | 9.34E-01 |
| m.961T>G | *RNR1* | 5 | rs3888511 | Non-coding |  | 2.50 (1.00-6.20) | 4.88E-02 | 2.03 (0.71-5.75) | 1.84E-01 |
| m.1811A>G | *RNR2* | 71 | rs28358576 | Non-coding | | 2.13 (1.06-4.25) | 3.32E-02 | 1.76 (0.61-5.09) | 3.00E-01 |
| m.2158T>C | *RNR2* | 5 | rs41349444 | Non-coding | | 3.22 (1.19-8.72) | 2.11E-02 | NA |  |
| m.2623A>G | *RNR2* | 10 |  | Non-coding | | 2.55 (1.24-5.22) | 1.07E-02 | 2.55 (1.21-5.39) | 1.38E-02 |
| m.3010G>A | *RNR2* | 129 | rs3928306 | Non-coding | | 0.65 (0.43-0.98) | 4.06E-02 | 0.78 (0.5-1.23) | 2.93E-01 |
| m.3197T>C | *RNR2* | 51 | rs2854131 | Non-coding | | 0.28 (0.09-0.89) | 3.12E-02 | 1.05 (0.19-5.78) | 9.56E-01 |
| m.10463T>C | *TR* | 57 | rs28358279 | Non-coding | | 0.22 (0.05-0.92) | 3.79E-02 | 0.31 (0.08-1.16) | 8.17E-02 |

D-Loop: control region. Models are adjusted for age at transplantation, IPSS-R score, MDS type, pre-HCT treatments and first 10 principal components.

*Significant *P* values after Bonferroni correction.

**Supplementary Table 5. Associations between common variants and relapse.**

| **Variant** | **Gene** | **N** | **dbSNP** | **Type** | **Amio Acid Change** | **Multivariable analysis** | | **Conditional analysis** | | **Fine-Gray Model** | |
| --- | --- | --- | --- | --- | --- | --- | --- | --- | --- | --- | --- |
|  |  |  |  |  |  | **HR (95% CI)** | ***P*** | **HR (95% CI)** | ***P*** | **sHR** | ***P*** |
| m.8697G>A | *ATP6* | 57 |  | Synonymous | M57M | 5.61 (1.4-22.43) | 1.48E-02 | 6.94 (1.6-30.12) | 9.72E-03 | 8.15 | 1.30E-04 |
| m.8843T>C | *ATP6* | 6 | rs386829053 | Missense | I106T | 3.74 (1.48-9.49) | 5.46E-03 | 3.06 (1.17-8.01) | 2.23E-02 | 4.60 | 5.70E-04 |
| m.16129G>A | *D-Loop* | 66 | rs41534744 | Non-coding | | 0.56 (0.33-0.94) | 2.77E-02 | 0.44 (0.25-0.77) | 4.25E-03 | 0.55 | 5.00E-02 |
| m.225G>A | *D-Loop* | 5 | rs372946833 | Non-coding | | 4.64 (1.32-16.32) | 1.68E-02 | 5.59 (1.37-22.7) | 1.62E-02 | 2.52 | 4.40E-01 |
| m.242C>T | *D-Loop* | 5 | rs1556422403 | Non-coding | | 4.98 (1.35-18.44) | 1.61E-02 | 5.58 (1.45-21.48) | 1.25E-02 | 3.17 | 8.00E-02 |
| m.538A>C | *D-Loop* | 15 | rs370922031 | Non-coding | | 0.31 (0.1-0.97) | 4.36E-02 | 0.33 (0.1-1.07) | 6.41E-02 | 0.33 | 3.60E-02 |
| m.15607A>G | *CYB* | 57 | rs193302996 | Synonymous | K287K | 18.61 (3.38-102.35) | 7.75E-04 | 25.49 (4.97-130.59) | 1.03E-04* | 22.44 | 1.60E-04 |
| m.5495T>C | *ND2* | 9 | rs3020602 | Synonymous | F342F | 3.82 (1.19-12.23) | 2.38E-02 | 4.67 (1.45-15.04) | 9.93E-03 | 2.20 | 2.50E-01 |
| m.13263A>G | *ND5* | 6 | rs28359175 | Synonymous | Q309Q | 5.24 (1.54-17.83) | 8.03E-03 | 4.33 (1.21-15.54) | 2.43E-02 | 4.19 | 8.10E-02 |
| m.2158T>C | *RNR2* | 5 | rs41349444 | Non-coding | | 4.98 (1.35-18.44) | 1.61E-02 | NA |  |  |  |
| m.2623A>G | *RNR2* | 10 |  | Non-coding | | 2.92 (1.17-7.3) | 2.21E-02 | 2.38 (0.91-6.24) | 7.82E-02 | 2.29 | 6.70E-02 |
| m.3010G>A | *RNR2* | 129 | rs3928306 | Non-coding | | 0.55 (0.32-0.95) | 3.30E-02 | 0.54 (0.3-0.96) | 3.55E-02 | 0.58 | 8.90E-02 |
| m.7465A>AC | *TS1* | 5 |  | Non-coding | | 4.08 (1.2-13.88) | 2.43E-02 | 4.16 (1.22-14.17) | 2.25E-02 | 5.32 | 4.60E-02 |

D-Loop: control region. Models are adjusted for age at transplantation, IPSS-R score, MDS type, pre-HCT treatments and first 10 principal components.

sHR: subdistribution hazard ratio.

*Significant *P* values after Bonferroni correction.

**Supplementary Table 6. Associations between common variants and transplant-related mortality.**

| **Variant** | **Gene** | **N** | **dbSNP** | **Type** | **Amio Acid Change** | **Multivariable analysis** | | **Conditional analysis** | | **Fine-Gray Model** | |
| --- | --- | --- | --- | --- | --- | --- | --- | --- | --- | --- | --- |
|  |  |  |  |  |  | **HR (95% CI)** | ***P*** | **HR (95% CI)** | ***P*** | **sHR** | ***P*** |
| m.8697G>A | *ATP6* | 57 |  | Synonymous | M57M | 0.10 (0.01-0.79) | 2.94E-02 | 1.00 (0.06-15.73) | 9.99E-01 | 0.13 | 1.40E-02 |
| m.8448T>C | *ATP8* | 5 | rs879056797 | Missense | M28T | 4.13 (1.25-13.63) | 2.01E-02 | NA |  |  |  |
| m.8512A>G | *ATP8* | 5 | rs1556423477 | Synonymous | K49K | 4.25 (1.25-14.50) | 2.08E-02 | 4.87 (1.39-17.02) | 1.31E-02 | 4.21 | 2.00E-02 |
| m.9656T>C | *CO3* | 6 | rs1556423706 | Synonymous | S150S | 9.25 (3.5-24.43) | 7.16E-06 | 14.35 (2.80-73.53) | 1.39E-03* | 12.35 | 8.60E-06 |
| m.16051A>G | *D-Loop* | 8 | rs117565943 | Non-coding | | 4.00 (1.36-11.73) | 1.16E-02 | 2.35 (0.57-9.72) | 2.38E-01 | 4.14 | 2.90E-02 |
| m.16255G>A | *D-Loop* | 5 | rs1603225769 | Non-coding | | 3.74 (1.05-13.30) | 4.13E-02 | 2.85 (0.79-10.28) | 1.09E-01 | 4.55 | 1.40E-02 |
| m.16260C>T | *D-Loop* | 6 | rs373855397 | Non-coding | | 3.97 (1.16-13.58) | 2.78E-02 | 2.02 (0.47-8.71) | 3.45E-01 | 3.92 | 4.60E-02 |
| m.16293A>G | *D-Loop* | 11 |  | Non-coding | | 3.47 (1.47-8.19) | 4.58E-03 | 1.56 (0.48-5.09) | 4.62E-01 | 2.32 | 5.90E-02 |
| m.16343A>G | *D-Loop* | 6 | rs374065731 | Non-coding | | 4.37 (1.30-14.73) | 1.74E-02 | NA |  |  |  |
| m.189A>G | *D-Loop* | 25 | rs371543232 | Non-coding | | 0.22 (0.05-0.96) | 4.38E-02 | 0.40 (0.10-1.65) | 2.06E-01 | 0.20 | 1.30E-01 |
| m.204T>C | *D-Loop* | 60 | rs3135032 | Non-coding | | 0.24 (0.10-0.59) | 1.69E-03 | 0.32 (0.13-0.77) | 1.13E-02 | 0.18 | 1.90E-03 |
| m.15454T>C | *CYB* | 6 | rs879015290 | Synonymous | L236L | 4.37 (1.30-14.73) | 1.74E-02 | NA |  |  |  |
| m.3590T>A | *ND1* | 139 |  | Missense | L95Q | 1.85 (1.03-3.33) | 4.06E-02 | 2.17 (1.16-4.05) | 1.56E-02* | 1.92 | 3.80E-02 |
| m.13762T>G | *ND5* | 472 | rs879154715 | Missense | S476A | 3.46 (1.10-10.94) | 3.41E-02 | 2.84 (0.91-8.88) | 7.32E-02 | 2.91 | 1.40E-01 |
| m.14139A>G | *ND5* | 6 | rs878918283 | Synonymous | L601L | 4.37 (1.30-14.73) | 1.74E-02 | 0.33 (0.04-2.81) | 3.09E-01 | 4.53 | 7.20E-02 |
| m.14323G>A | *ND6* | 6 | rs879208488 | Synonymous | N117N | 3.67 (1.06-12.68) | 3.95E-02 | 3.33 (0.88-12.62) | 7.63E-02 | 4.64 | 6.60E-02 |
| m.14587A>G | *ND6* | 5 | rs1556424469 | Synonymous | G29G | 2.80 (1.00-7.84) | 4.98E-02 | 1.05 (0.33-3.37) | 9.37E-01 | 3.90 | 4.80E-06 |
| m.961T>G | *RNR1* | 5 | rs3888511 | Non-coding | | 4.13 (1.25-13.63) | 2.01E-02 | 4.66 (0.86-25.14) | 7.38E-02 | 3.43 | 2.60E-02 |
| m.1811A>G | *RNR2* | 71 | rs28358576 | Non-coding | | 3.46 (1.32-9.09) | 1.19E-02 | 1.63 (0.44-5.98) | 4.60E-01 | 4.94 | 1.80E-03 |
| m.10463T>C | *TR* | 57 | rs28358279 | Non-coding | | 0.09 (0.01-0.60) | 1.31E-02 | 0.18 (0.01-2.68) | 2.12E-01 | 0.14 | 2.30E-02 |

Models are adjusted for age at transplantation, IPSS-R score, MDS type, pre-HCT treatments and first 10 principal components.

sHR: subdistribution hazard ratio.

*Significant *P* values after Bonferroni correction.

**Supplementary Table 7. Associations between mitochondrial genes and MDS outcomes after allo-HCT.**

| **Gene** | **No. of MDS** | **No. of Death** | **No. of Relapse** | **No. of TRM** | **OS** | **RFS** | **Relapse** | **TRM** |
| --- | --- | --- | --- | --- | --- | --- | --- | --- |
| **Control region** | 490 | 277 | 177 | 135 | 0.05 | 0.25 | 0.40 | 0.16 |
| **Complex I** |  |  |  |  |  |  |  |  |
| *ND1* | 410 | 237 | 150 | 117 | 0.17 | 0.42 | 0.03 | 0.11 |
| *ND2* | 482 | 270 | 173 | 133 | 3.06×10^-3^* | 0.01 | 5.92×10^-4^* | 0.14 |
| *ND3* | 159 | 98 | 61 | 49 | 0.38 | 0.19 | 0.11 | 0.52 |
| *ND4* | 351 | 202 | 132 | 99 | 1.41×10^-3^* | 7.75×10^-4^* | 0.01 | 0.01 |
| *ND4L* | 104 | 55 | 49 | 20 | 0.01 | 0.08 | 0.26 | 4.91×10^-4^* |
| *ND5* | 487 | 277 | 177 | 135 | 0.23 | 0.07 | 1.91×10^-3^* | 0.04 |
| *ND6* | 201 | 115 | 77 | 56 | 0.18 | 0.09 | 0.12 | 0.32 |
| **Complex III** |  |  |  | 134 |  |  |  |  |
| *CYB* | 488 | 275 | 176 | 134 | 1.04×10^-3^* | 2.87×10^-3^* | 7.03×10^-4^* | 2.24×10^-3^* |
| **Complex IV** |  |  |  |  |  |  |  |  |
| *CO1* | 401 | 237 | 148 | 116 | 0.23 | 0.05 | 0.43 | 0.13 |
| *CO2* | 148 | 76 | 44 | 43 | 0.04 | 0.02 | 0.13 | 0.13 |
| *CO3* | 232 | 138 | 90 | 63 | 0.10 | 0.88 | 0.37 | 0.68 |
| **Complex V** |  |  |  |  |  |  |  |  |
| *ATP6* | 490 | 276 | 177 | 135 | 0.03 | 0.01 | 0.20 | 0.12 |
| *ATP8* | 59 | 40 | 23 | 23 | 0.04 | 0.02 | 0.60 | 0.03 |
| **rRNA** | 489 | 276 | 176 | 135 | 0.27 | 4.15×10^-3^ | 0.20 | 0.69 |
| **tRNA** | 331 | 189 | 117 | 96 | 0.08 | 0.03 | 1.99×10^-4^* | 0.06 |

Models are adjusted for age at transplantation, IPSS-R score, MDS type, pre-HCT treatments and first 10 principal components.

*Significant *P* values after Bonferroni correction.

**Supplementary Table 8. *P* values of gene-based analysis with Burden test and SKAT.**

| **Gene** | **Burden Test** | | | | **SKAT** | | | |
| --- | --- | --- | --- | --- | --- | --- | --- | --- |
|  | **OS** | **RFS** | **Relapse** | **TRM** | **OS** | **RFS** | **Relapse** | **TRM** |
| **D-Loop** | 0.30 | 0.34 | 0.45 | 0.57 | 0.18 | 0.22 | 0.27 | 0.09 |
| **Complex I** |  |  |  |  |  |  |  |  |
| *ND1* | 0.11 | 0.23 | 0.44 | 0.37 | 2.62E-04* | 1.52E-03* | 0.12 | 2.39E-05* |
| *ND2* | 0.20 | 0.47 | 0.39 | 0.88 | 0.01 | 0.09 | 0.19 | 0.00 |
| *ND3* | 0.84 | 0.73 | 0.84 | 0.78 | 0.45 | 0.37 | 0.03 | 0.70 |
| *ND4* | 0.76 | 0.15 | 0.08 | 0.86 | 0.01 | 0.02 | 1.95E-04* | 0.02 |
| *ND4L* | 0.08 | 0.57 | 0.14 | 0.02 | 0.06 | 0.05 | 0.16 | 0.22 |
| *ND5* | 0.03 | 0.03 | 0.09 | 0.15 | 0.04 | 0.08 | 0.04 | 8.18E-03 |
| *ND6* | 0.91 | 0.93 | 0.97 | 0.84 | 6.28E-03 | 0.03 | 7.30E-03 | 0.02 |
| **Complex** III |  |  |  |  |  |  |  |  |
| *CYB* | 0.65 | 0.69 | 0.85 | 0.70 | 0.08 | 0.12 | 0.18 | 3.27E-03 |
| **Complex** IV |  |  |  |  |  |  |  |  |
| *CO1* | 0.04 | 0.09 | 0.23 | 0.25 | 0.01 | 4.46E-03 | 5.94E-04* | 5.70E-04* |
| *CO2* | 0.53 | 0.59 | 0.20 | 0.49 | 0.02 | 0.09 | 0.19 | 2.51E-03* |
| *CO3* | 0.01 | 0.03 | 0.05 | 0.32 | 8.04E-03 | 0.07 | 0.02 | 4.34E-05* |
| **Complex** V |  |  |  |  |  |  |  |  |
| *ATP6* | 0.62 | 0.96 | 0.96 | 0.87 | 0.14 | 0.21 | 0.02 | 0.02 |
| *ATP8* | 0.02 | 2.48E-03* | 0.12 | 6.41E-03 | 0.02 | 0.01 | 0.27 | 0.02 |
| **rRNA** | 0.72 | 0.77 | 0.83 | 0.51 | 0.07 | 0.15 | 0.05 | 7.39E-04* |
| **tRNA** | 0.45 | 0.37 | 0.77 | 0.10 | 0.06 | 0.03 | 0.01 | 1.24E-04* |

Models are adjusted for age at transplantation, IPSS-R score, MDS type, pre-HCT treatments and first 10 principal components.

*Significant *P* values after Bonferroni correction.

**Supplementary Table 9. Associations between mitochondrial haplogroups and MDS outcomes after allo-HCT.**

| **Haplogroup** | **No.** | **OS** | | **RFS** | | **Relapse** | | **TRM** | |
| --- | --- | --- | --- | --- | --- | --- | --- | --- | --- |
|  |  | HR (95%CI) | *P* | HR (95%CI) | *P* | HR (95%CI) | *P* | HR (95%CI) | *P* |
| H | 215 | **Ref.** | | | | | | | |
| U | 71 | 0.88 (0.60-1.31) | 0.54 | 0.79 (0.54-1.15) | 0.22 | 0.72 (0.44-1.20) | 0.21 | 0.88 (0.50-1.55) | 0.66 |
| J | 52 | 1.23 (0.83-1.83) | 0.31 | 1.30 (0.90-1.89) | 0.17 | 1.09 (0.64-1.83) | 0.76 | 1.59 (0.94-2.72) | 0.08 |
| T | 50 | 1.16 (0.78-1.72) | 0.47 | 1.29 (0.89-1.86) | 0.18 | 1.27 (0.78-2.07) | 0.34 | 1.30 (0.74-2.29) | 0.36 |
| K | 40 | 1.21 (0.78-1.89) | 0.39 | 1.43 (0.96-2.14) | 0.08 | **1.70 (1.03-2.81)** | **0.04** | 1.08 (0.55-2.14) | 0.83 |
| V | 16 | 1.29 (0.67-2.48) | 0.45 | 1.00 (0.52-1.92) | 0.99 | 0.86 (0.35-2.15) | 0.75 | 1.17 (0.46-2.98) | 0.75 |
| I | 13 | **2.32 (1.23-4.35)** | **0.01** | **2.04 (1.09-3.83)** | **0.03** | 1.77 (0.76-4.13) | 0.19 | 2.41 (0.95-6.14) | 0.06 |
| W | 13 | 0.81 (0.35-1.85) | 0.61 | 0.66 (0.29-1.50) | 0.32 | 0.53 (0.17-1.69) | 0.29 | 0.85 (0.26-2.72) | 0.78 |
| X | 8 | 1.16 (0.47-2.86) | 0.75 | 1.58 (0.73-3.43) | 0.24 | 1.66 (0.60-4.62) | 0.33 | 1.50 (0.46-4.90) | 0.50 |
| N | 8 | 0.90 (0.37-2.24) | 0.83 | 1.01 (0.44-2.31) | 0.99 | 0.89 (0.28-2.90) | 0.85 | 1.15 (0.35-3.76) | 0.81 |
| A/B/C/M/R/Z | 8 | 1.45 (0.58-3.60) | 0.43 | 1.58 (0.69-3.64) | 0.28 | 1.75 (0.62-4.91) | 0.29 | 1.31 (0.31-5.44) | 0.71 |

Models are adjusted for age at transplantation, IPSS-R score, MDS type and pre-HCT treatments.

Bold represent significant associations.

**Supplementary Table 10. C-index of the models in patients with wild-type *TP53*.**

| **Model** | **OS** | **RFS** | **Relapse** | **TRM** |
| --- | --- | --- | --- | --- |
| IPSS-R | 0.45 | 0.44 | 0.43 | 0.38 |
| Clinical | 0.55 | 0.53 | 0.53 | 0.52 |
| mtDNA | 0.59 | 0.59 | 0.63 | 0.59 |
| IPSS-R + mtDNA | 0.63 | 0.63 | 0.65 | 0.63 |
| Clinical + mtDNA | 0.66 | 0.65 | 0.68 | 0.66 |

Clinical model includes IPSS-R, MDS type and pre-transplantation treatments.

**Supplementary Table 11. Clinical variables selected for overall survival.**

| **Variable** | **Beta** | **HR** | **SE** | ***P*-value** |
| --- | --- | --- | --- | --- |
| IPSS-R = intermediate | 0.31 | 1.36 | 0.15 | 0.04 |
| IPSS-R = high/very high | 0.71 | 2.03 | 0.17 | 1.91E-05 |
| MDS type = therapy related | -0.12 | 0.89 | 0.15 | 0.41 |
| MDS type = secondary | -0.73 | 0.48 | 0.46 | 0.11 |
| HMA = Yes | 0.29 | 1.33 | 0.16 | 0.07 |
| CHEMO = Yes | 0.29 | 1.34 | 0.20 | 0.14 |

Reference groups: IPSS-R = low/very low, MDS-type = *de novo*, HMA = none, CHEMO = none

Variables selected for adjustment in the models via a forward-backward variable selection.
