# Supplementary material for: Prognostic landscape of mitochondrial genome in myelodysplastic syndrome after stem-cell transplantation
Source: J Hematol Oncol. 2023 Mar 10;16:21. doi: 10.1186/s13045-023-01418-4 (PMC9999628; doi:10.1186/s13045-023-01418-4)
Supplement: Supplementary file 3 — Additional file 3. Supplementary Methods. [file 13045_2023_1418_MOESM3_ESM.docx]

**Supplementary Methods**

**Data Source**

The CIBMTR is a research affiliation between the National Marrow Donor Program/Be The Match and the Medical College of Wisconsin. It facilitates critical research through medical, scientific, and statistical expertise; a network of more than 350 participating centers worldwide; an outcomes database with clinical data on more than 575,000 patients; and a biospecimen repository [1]. The study was approved by the Institutional Review Board of CIBMTR and conducted in accordance with the Declaration of Helsinki.

**Patient Cohort and DNA Sequencing**

Patients with MDS who underwent allo-HCT between 2014 and 2018 were enrolled from the CIBMTR repository and research database. Patients were excluded if they were diagnosed with AML (percentage of blasts in the bone marrow or blood ≥ 20%) or if they had received a diagnosis of chronic myelomonocytic leukemia or overlap myelodysplastic-myeloproliferative neoplasms. A total of 494 eligible patients who had whole blood samples cryopreserved were selected for WGS. The median age of our cohort was 67 (range, 23-79) years, the median KPS was 80, and 180 patients (36.4%) had poor/very poor IPSS-R scores. Seventy one percent of patients in the cohort received transplants from well matched (8/8) unrelated donors, and 26% received myeloablative conditioning regimens. All patients were of European ancestry. The median follow-up among survivors was 34.5 months (range 3.2 - 62.7) (Supplementary Table 1).

All sequences were generated by Illumina NovaSeq 6000 platform (Illumina) with 150-bp paired-end run. A detailed description of the sample preparation, quality control (QC) and data processing have been previously reported [2].

**Mitochondrial Genome Analysis**

Mitochondrial genome sequences were extracted from the WGS data and analyzed using MToolBox pipeline (v1.2.1) [3, 4]. All reads were aligned to the mtDNA reference genome (Revised Cambridge Reference Sequence, rCRS) (GeneBank accession number: NC_012920) and the human reference genome (GRCh38) by GSNAP (v2015-12-31) to discard nuclear mitochondrial sequences and amplification artifacts. On average, the sequencing depth of the mitochondrial genome was 14,596× (213× - 17,070×), which was much higher than that of the nuclear genome (77×) [2], allowing for confident detection of mutations with low heteroplasmic levels (Supplementary Figure 9). The average coverage for the mitochondrial genome was 99.90% (99.89% - 100%). Reads with putative indels were realigned locally using 127 known indels annotated in the mtDNA databases HmtDB and MITOMAP [5, 6]. Putative mtDNA variants, including single nucleotide variants (SNV) and indels, were identified by parsing the SAM CIGAR string. Heteroplasmic fraction (HF, %) was assessed as the fraction of the variant read depth onto the total mitochondrial read depth of the same position (for mismatches and deletions) or of the 5’ flanking position (for insertions), for each variant allele passing standard QC. We considered variants with HF > 90% as homoplasmic (variants affecting all the mtDNA copies within a cell compared to a standard sequence), HF between 10% and 90% as heteroplasmic (the presence of a mixture of more than one type of mtDNA within a cell), and HF < 10% as low heteroplasmic. Additional QC was conducted for mtDNA variants, including filtering out variants with low base quality score (QS < 25), low coverage (< 100 read depth), HF <1% or had “artifact_prone_site” and “indel_stack” flags in the gnomAD v3.1 database [7]. A fragment-classify tool was used for haplogroup prediction [5]. This tool aligns each sample-specific reconstructed contig against the related macro haplogroup-specific consensus sequence (MHCS) to recognize variants. To discover the mutational signatures, a hierarchical Dirichlet process and SigProfiler workflow were used to extract both known and *de novo* mutational signatures [8, 9]. All extracted processes were assigned to one specific mutational signature included in the updated COSMIC database (release v93, March 2021) [10].

To predict the pathogenicity of mtDNA variants, we integrated information from different databases. Variants, regardless of their biotype, were classified as “pathogenic” if they (1) were listed as “confirmed” pathogenic in MITOMAP database [6], (2) had clinical significance of “pathogenic/likely pathogenic” based on the modified American College of Medical Genetics and Genomics and Association of Molecular Pathology (ACMG/AMP) guidelines for mtDNA variants (criteria provided in ClinVar) [11], or (3) identified as loss of function (LoF) variants by the Ensembl Variant Effect Predictor (VEP) [12]. According to the mtDNA specifications of the ACMG/AMP guidelines, mtDNA variants with allele frequency in the population database < 0.00002 provide evidence of pathogenicity [13]. Based on this criterion, variants with AF_hom < 0.00002 in the gnomAD v3.1 database were also considered as “pathogenic” if they were non-synonymous and predicted to be deleterious from 5 of 7 prediction tools (HmtVar, SIFT, PolyPhen-2, MutPred2, PANTHER, PhD-SNP and SNPs&GO) [14-20], or had MToolBox disease score > 0.4311 [21]. For *MT-tRNA* and *MT-rRNA,* variants with AF_hom < 0.00002 were considered “pathogenic” if predicted to be deleterious from 3 of 4 prediction tools (HmtVar, MitoTIP, PON-mt-tRNA and Mamit-tRNA) [6, 14, 22, 23], or had MToolBox RNA prediction score ≥ 0.31 for *MT-tRNAs* and ≥ 0.51 for *MT-rRNAs* [24-26].

**Statistical Analysis**

Associations between mtDNA mutational count (0 and 1+) and patient clinical characteristics were estimated using logistic regression for age at transplant, and χ2 test for KPS, IPSS-R score, MDS types and individual cytogenetic abnormalities. Overall survival (OS) was defined as the time from transplantation until death from any cause or last follow-up. Relapse-free survival (RFS) was defined as time to relapse, or death in first 28 days from any cause, or death in remission from any cause after 28 days. We analyzed OS and RFS using the Kaplan-Meier method and log-rank tests. We also estimated hazard ratios (HRs) and 95% CIs using a Cox proportional hazard model. Relapse definition was based on evidence of disease recurrence post HCT, with death in remission being a competing risk. Transplant-related mortality (TRM) was defined as death in remission, with relapse being a competing risk. Fine and Gray competing risks regression models were applied to analyze time to TRM and time to relapse events. To identify clinical variables for model adjustment, we applied a forward-backward variable selection to test the prognostic importance of clinical variables listed in Supplementary Table 11 for OS. IPSS-R, pre-transplant treatments, and MDS type were identified to be important and included in the final model [2]. To control the possible presence of population stratification, we calculated principal components (PCs) using the complete mtDNA sequencing data. Common variants with allele frequency (AF) > 1% were tested for associations with transplantation outcomes individually. Candidate variants with *P* values < 0.05 was further selected for a conditional analysis with adjustment for all other candidate variants. The cumulative effects of mutations in mitochondrial genes were evaluated using the optimized sequence kernel association test (SKAT-O), which combines the burden test and SKAT with small sample size adjustment [27]. Positions of mitochondrial genes and non-coding regions were obtained from MITOMAP database [6]. Concordance indices (C-index) were calculated with a non-parametric bootstrap procedure (number of bootstraps=100) using a random survival forest (RSF) model to estimate the prognostic performance of mtDNA mutations in post allo-HCT outcomes. We further quantified the clinical impact of mtDNA mutations by reclassification using the net reclassification index (NRI) [28]. Survival curves were constructed using the Kaplan-Meier method and compared using the log-rank test. Significance was determined at a two-sided α level of 0.05 and Bonferroni correction was applied to account for multiple testing. All statistical analyses were conducted in the R environment (v4.0.2).

**REFERENCES**

1. CIBMTR 2020 annual report [Available from: <https://www.cibmtr.org/About/AdminReports/Documents/2020CIBMTRAnnualReport.pdf>.

2. Zhang T, Auer P, Spellman SR, Fretham C, Saber W, Bolon Y-T. Genomic Subgroups Impact Post-Transplant Survival in Patients with Myelodysplastic Syndrome: A CIBMTR Analysis. Blood. 2021;138(Supplement 1):3678-.

3. Calabrese C, Simone D, Diroma MA, Santorsola M, Gutta C, Gasparre G, et al. MToolBox: a highly automated pipeline for heteroplasmy annotation and prioritization analysis of human mitochondrial variants in high-throughput sequencing. Bioinformatics. 2014;30(21):3115-7.

4. Picardi E, Pesole G. Mitochondrial genomes gleaned from human whole-exome sequencing. Nature methods. 2012;9(6):523-4.

5. Rubino F, Piredda R, Calabrese FM, Simone D, Lang M, Calabrese C, et al. HmtDB, a genomic resource for mitochondrion-based human variability studies. Nucleic Acids Res. 2012;40(Database issue):D1150-9.

6. Lott MT, Leipzig JN, Derbeneva O, Xie HM, Chalkia D, Sarmady M, et al. mtDNA Variation and Analysis Using Mitomap and Mitomaster. Curr Protoc Bioinformatics. 2013;44:1 23 1-6.

7. Karczewski KJ, Francioli LC, Tiao G, Cummings BB, Alföldi J, Wang Q, et al. The mutational constraint spectrum quantified from variation in 141,456 humans. Nature. 2020;581(7809):434-43.

8. Maura F, Degasperi A, Nadeu F, Leongamornlert D, Davies H, Moore L, et al. A practical guide for mutational signature analysis in hematological malignancies. Nature communications. 2019;10(1):2969.

9. Alexandrov LB, Kim J, Haradhvala NJ, Huang MN, Tian Ng AW, Wu Y, et al. The repertoire of mutational signatures in human cancer. Nature. 2020;578(7793):94-101.

10. Tate JG, Bamford S, Jubb HC, Sondka Z, Beare DM, Bindal N, et al. COSMIC: the Catalogue Of Somatic Mutations In Cancer. Nucleic Acids Res. 2019;47(D1):D941-D7.

11. Landrum MJ, Lee JM, Benson M, Brown GR, Chao C, Chitipiralla S, et al. ClinVar: improving access to variant interpretations and supporting evidence. Nucleic Acids Res. 2018;46(D1):D1062-D7.

12. McLaren W, Gil L, Hunt SE, Riat HS, Ritchie GR, Thormann A, et al. The Ensembl Variant Effect Predictor. Genome Biol. 2016;17(1):122.

13. McCormick EM, Lott MT, Dulik MC, Shen L, Attimonelli M, Vitale O, et al. Specifications of the ACMG/AMP standards and guidelines for mitochondrial DNA variant interpretation. Hum Mutat. 2020;41(12):2028-57.

14. Preste R, Vitale O, Clima R, Gasparre G, Attimonelli M. HmtVar: a new resource for human mitochondrial variations and pathogenicity data. Nucleic Acids Res. 2019;47(D1):D1202-D10.

15. Vaser R, Adusumalli S, Leng SN, Sikic M, Ng PC. SIFT missense predictions for genomes. Nature protocols. 2016;11(1):1-9.

16. Adzhubei IA, Schmidt S, Peshkin L, Ramensky VE, Gerasimova A, Bork P, et al. A method and server for predicting damaging missense mutations. Nature methods. 2010;7(4):248-9.

17. Pejaver V, Urresti J, Lugo-Martinez J, Pagel KA, Lin GN, Nam HJ, et al. Inferring the molecular and phenotypic impact of amino acid variants with MutPred2. Nature communications. 2020;11(1):5918.

18. Mi H, Ebert D, Muruganujan A, Mills C, Albou LP, Mushayamaha T, et al. PANTHER version 16: a revised family classification, tree-based classification tool, enhancer regions and extensive API. Nucleic Acids Res. 2021;49(D1):D394-D403.

19. Capriotti E, Calabrese R, Casadio R. Predicting the insurgence of human genetic diseases associated to single point protein mutations with support vector machines and evolutionary information. Bioinformatics. 2006;22(22):2729-34.

20. Calabrese R, Capriotti E, Fariselli P, Martelli PL, Casadio R. Functional annotations improve the predictive score of human disease-related mutations in proteins. Hum Mutat. 2009;30(8):1237-44.

21. Santorsola M, Calabrese C, Girolimetti G, Diroma MA, Gasparre G, Attimonelli M. A multi-parametric workflow for the prioritization of mitochondrial DNA variants of clinical interest. Human genetics. 2016;135(1):121-36.

22. Niroula A, Vihinen M. PON-mt-tRNA: a multifactorial probability-based method for classification of mitochondrial tRNA variations. Nucleic Acids Res. 2016;44(5):2020-7.

23. Putz J, Dupuis B, Sissler M, Florentz C. Mamit-tRNA, a database of mammalian mitochondrial tRNA primary and secondary structures. RNA. 2007;13(8):1184-90.

24. Smith PM, Elson JL, Greaves LC, Wortmann SB, Rodenburg RJ, Lightowlers RN, et al. The role of the mitochondrial ribosome in human disease: searching for mutations in 12S mitochondrial rRNA with high disruptive potential. Human molecular genetics. 2014;23(4):949-67.

25. Yarham JW, Al-Dosary M, Blakely EL, Alston CL, Taylor RW, Elson JL, et al. A comparative analysis approach to determining the pathogenicity of mitochondrial tRNA mutations. Hum Mutat. 2011;32(11):1319-25.

26. Blakely EL, Yarham JW, Alston CL, Craig K, Poulton J, Brierley C, et al. Pathogenic mitochondrial tRNA point mutations: nine novel mutations affirm their importance as a cause of mitochondrial disease. Hum Mutat. 2013;34(9):1260-8.

27. Lee S, Wu MC, Lin X. Optimal tests for rare variant effects in sequencing association studies. Biostatistics. 2012;13(4):762-75.

28. Leening MJ, Vedder MM, Witteman JC, Pencina MJ, Steyerberg EW. Net reclassification improvement: computation, interpretation, and controversies: a literature review and clinician's guide. Ann Intern Med. 2014;160(2):122-31.
